# Emodin and the Anthraquinone Scaffold: Therapeutic Promise and Strategies to Overcome Translational Barriers

**DOI:** 10.3390/molecules31050833

**Published:** 2026-03-02

**Authors:** Rositsa Mihaylova, Viktoria Elincheva, Rumyana Simeonova, Georgi Momekov

**Affiliations:** Department of Pharmacology, Pharmacotherapy and Toxicology, Faculty of Pharmacy, Medical University of Sofia, 1000 Sofia, Bulgaria; v.lyubomirova@pharmfac.mu-sofia.bg (V.E.); gmomekov@pharmfac.mu-sofia.bg (G.M.)

**Keywords:** emodin, anthraquinones, aloe-emodin, chrysophanol, rhein, diacerein, physcion, cancer, immunomodulation, inflammation, nanoformulations

## Abstract

Emodin, a trihydroxy-methyl anthraquinone abundant in rhubarb, *Polygonum* species, and other medicinal plants, exemplifies the therapeutic potential and translational complexity of the broader anthraquinone scaffold. Anthraquinone derivatives have demonstrated antiproliferative, anti-inflammatory, metabolic, cardiovascular, antifibrotic, and immunomodulatory effects, consistently reported across diverse preclinical models, targeting pathways such as NF-κB, PI3K/AKT, MAPKs, AMPK, PPARs, NLRP3, and ferroptosis-related axes. Despite strong preclinical efficacy, clinical development has been limited by unfavorable absorption, distribution, metabolism, and excretion (ADME) characteristics, including poor aqueous solubility, extensive first-pass glucuronidation, and active efflux via intestinal and hepatic transporters. These features result in low and variable systemic exposure, while high local concentrations, particularly in the gastrointestinal tract, contribute to context-dependent toxicity signals that complicate risk assessment. The present review integrates pharmacological, toxicological, and formulation-focused evidence to provide a unified assessment of emodin and the anthraquinone scaffold. Particular emphasis is placed on bidirectional, dose- and context-dependent effects on the liver and kidney; the modulation of cytochrome P450 enzymes, UGTs, and transporters; and emerging preclinical formulation strategies that aim to decouple intrinsic bioactivity from pharmacokinetic limitations.

## 1. Introduction

Anthraquinones constitute a widely distributed phytochemical scaffold in medicinal plants (notably *Rheum* spp. and *Aloe* spp.) and have long attracted biomedical interest because modest substitutions on a redox-active quinone core can yield markedly divergent anti-inflammatory, anticancer, and metabolic properties [1]. Among these congeners, emodin (1,3,8-trihydroxy-6-methylanthraquinone) has emerged as the most intensively studied “hub” molecule, with consistent preclinical efficacy across tumor, inflammatory, and immunometabolic disease models, and a rapidly expanding formulation literature aimed at improving its drug-like behavior [2].

However, the same quinone/phenolic chemistry that underpins pleiotropic bioactivity also confers context-dependent liabilities, including oxidative stress, mitochondrial perturbation, and enzyme/transporter interactions, which complicate translation from cell systems to organism-level pharmacology and ultimately to clinical development [3]. In addition, clinical translation remains constrained by poor oral exposure and pronounced first-pass metabolism, with extensive glucuronidation and transporter-linked disposition shaping systemic availability and contributing to variability across dosing regimens and experimental models [4]. Toxicological reports further illustrate the “two-sided” nature of anthraquinone biology: emodin can induce hepatic and renal injury via mitochondrial dysfunction, ROS-linked stress responses, and bioactivation pathways that may generate potentially more reactive metabolites (e.g., 5-hydroxyemodin) under specific enzyme-induction contexts [5]. Accordingly, translational progress increasingly depends on formulational and chemical strategies such as nanoemulsions that may reduce presystemic metabolism and enhance systemic exposure, targeted micelles and liposomes that improve tissue delivery, and scaffold-guided derivatives with improved oral bioavailability, all designed to enhance efficacy while managing class liabilities [6].

The present review therefore revisits emodin as the exemplar of the anthraquinone scaffold, synthesizing evidence across therapeutic domains while also exploring similarities and differences with other related anthraquinones such as aloe-emodin, chrysophanol, rhein/diacerein, and physcion. In addition, the review contextualizes these compounds by summarizing their natural occurrence, major botanical source, and chemical forms present in traditional medicinal preparations and dietary matrices. We also focus on the central translational bottlenecks: structure–activity relationships (SARs), pharmacokinetics, safety signals, interaction potential, and delivery approaches, where rational engineering can convert robust preclinical promise into credible therapeutic trajectories.

## 2. Structural Features and SARs Among Natural Anthraquinones

**Figure 1:**
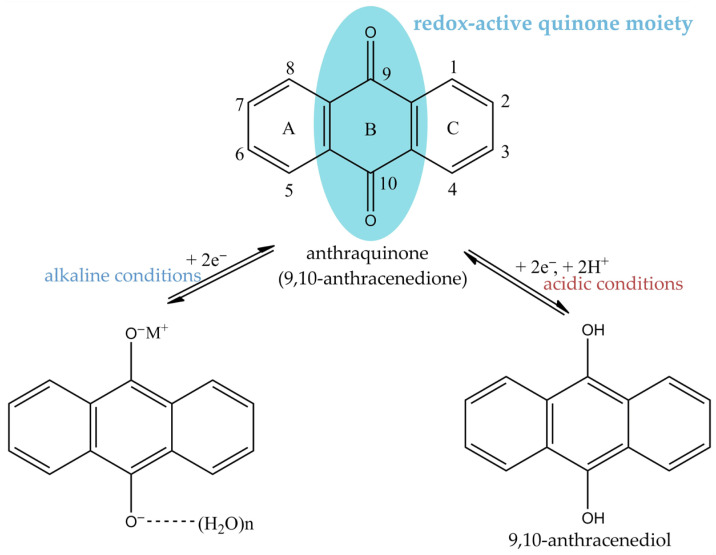
Structural and redox chemistry of the anthraquinone scaffold: core quinone moiety and redox cycling under acidic and alkaline conditions.

Emodin belongs to a closely related family of naturally occurring anthraquinones, built on the planar anthracene-9,10-dione scaffold. The presence of a rigid, polyaromatic quinone core, and multiple hydrogen-bonding groups is central to emodin’s biological behavior. The quinone moiety (two carbonyls at C9/C10) provides an electrophilic/redox-active center, while the phenolic -OH groups act as H-bond donors/acceptors that can stabilize binding to proteins and influence redox cycling and metal interactions [7,8,9,10]. Because of their conjugated double-bond system, anthraquinones absorb in both the UV and visible regions, typically showing λmax values around 204–212 and 248–254 nm that are characteristic of the anthraquinone chromophore. Changes in solution color under alkaline conditions are widely used for preliminary structural characterization: 1,8-dihydroxy-substituted anthraquinones appear red in basic medium, whereas anthrones and dianthrones are initially yellow but rapidly turn red as they are oxidized to the corresponding anthraquinones (Figure 1).

**Figure 2:**
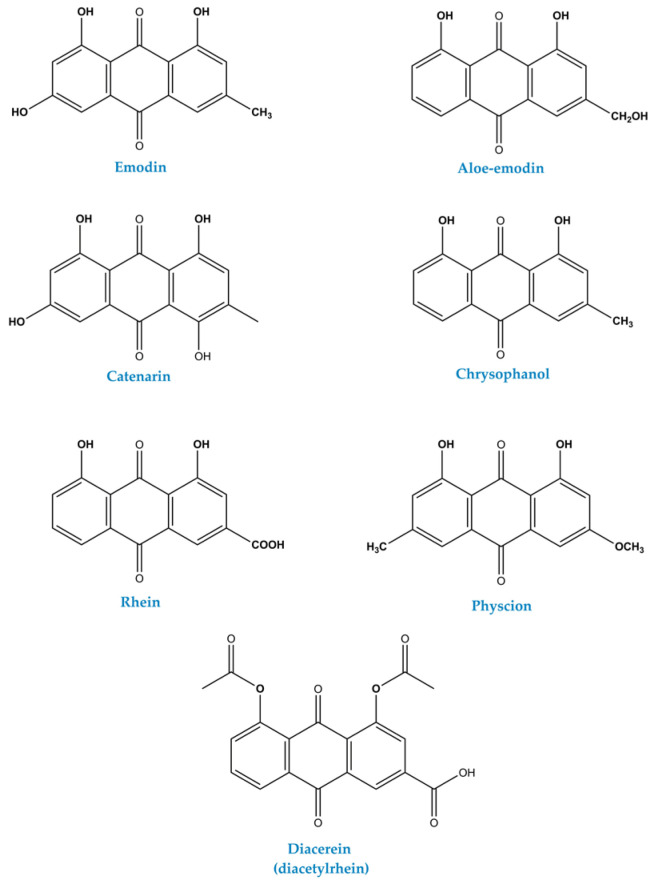
Chemical structures of emodin and representative naturally occurring and semi-synthetic anthraquinone derivatives.

Emodin, aloe-emodin, rhein, chrysophanol, and physcion share the same 9,10 anthracenedione core but differ at a small number of positions by methyl, hydroxymethyl, carboxyl, hydroxyl, or methoxy substituents (Figure 2). The available literature data across this structural family clearly indicate that subtle modifications of the anthraquinone scaffold can lead to pronounced differences in solubility, redox behavior, target engagement, and ADME profiles, with important implications for both pharmacology and toxicity [11,12]. These substituents modulate electron density and inter- and intramolecular hydrogen bonding across the π system, which in turn affects pKa values, solubility profiles, and redox properties in biologically relevant environments [7,13,14,15].

The representative anthraquinone derivative emodin, for example, bears three phenolic hydroxyl groups and a methyl substituent (6-methyl-1,3,8-trihydroxy-9,10-anthraquinone), whereas its closely related aloe-emodin analogue carries a hydroxymethyl group, introducing an additional H-bond donor/acceptor and increasing overall polarity. The increased aqueous compatibility and altered redox potential result in somewhat stronger antibacterial and often higher in vitro cytotoxic activities of aloe-emodin and rhein compared to more hydrophobic anthraquinone congeners [8,16]. Rhein introduces a carboxylic acid group into the same core, conferring pH-dependent ionization and markedly different aqueous behavior relative to neutral aglycones such as emodin and chrysophanol. Deprotonation of the carboxyl function at physiological pH greatly enhances solubility and alters plasma protein binding, which may increase systemic exposure while simultaneously reducing passive membrane permeability. In terms of target engagement, the more acidic rhein often exhibits distinct binding and potency profiles (e.g., in antibacterial and anti-inflammatory assays) compared with less polar congeners, demonstrating how the introduction of a single ionizable COOH group can substantially readjust target versus off-target interactions [16,17].

Conversely, chrysophanol and physcion are more hydrophobic natural anthraquinones with fewer phenolic hydroxyl groups than emodin, which decreases hydrogen bonding capacity and increases hydrophobicity. Physcion carries a methoxy substituent in place of a phenolic hydroxyl, a classic O-methylation that increases lipophilicity, reduces acidity, and removes one site susceptible to direct conjugative metabolism (e.g., glucuronidation) at that position. Increased lipophilicity favors membrane partitioning and may promote broader tissue distribution, shifting ADME toward distribution-driven rather than solubility limited behavior in vivo [18,19]. Existing SARs analyses indicate that electron-donating methoxy and methyl groups tend to weaken antibacterial potency compared to their hydroxyl or carboxyl analogues in certain bacterial assays. In addition, substituent type and position collectively determine redox reactivity and the antioxidant versus pro-oxidant behavior across this series of closely related anthraquinones [17,18].

## 3. Natural Occurrence and Major Sources

**Figure 3:**
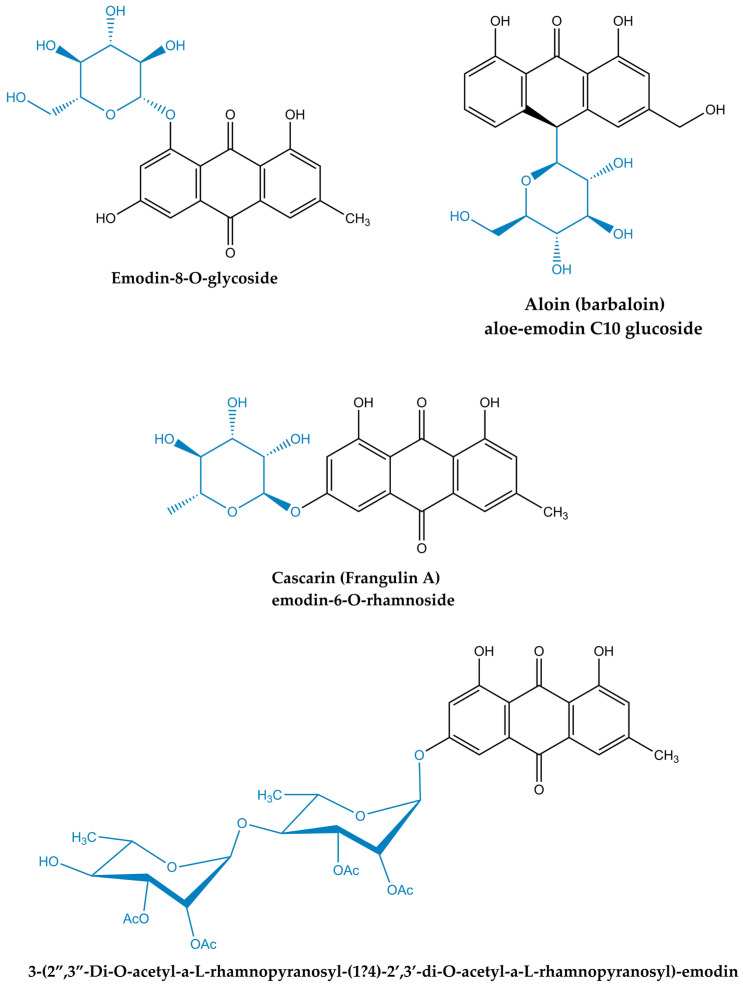
Plant-derived anthraquinone glycosides and related synthetic sugar conjugates based on the emodin scaffold.

In planta, emodin-related anthraquinones occur predominantly as O-glycosides and related sugar conjugates (e.g., emodin 8-O-glucoside, aloe-emodin-8-O-glucoside, chrysophanol-8-O-glucoside, physcion-8-O-glucoside), with free aglycones representing a smaller fraction (Figure 3) [8]. These glycosides primarily function as inactive storage forms of the aglycone and may act as pro-forms that regenerate emodin upon enzymatic and microbiota-driven hydrolysis. However, sugar conjugation does not necessarily abolish biological activity, as glycosides may also display intrinsic bioactivities distinct from those of the corresponding aglycone, depending on hydrolysis efficiency and biological context. For example, a well-studied member of the anthraquinone family in the context of osteoarthritis treatment is aloin, also known as barbaloin, a glycosylated derivative of aloe-emodin found in many species of the *Aloe* genus [8]. Given the markedly higher aqueous solubility of the glycosylated anthraquinone forms, they also exhibit distinct intestinal absorption, metabolism, and pharmacokinetic profiles shaped by variabilities in gut flora and phase II metabolism [20]. This is of particular relevance for their biological evaluation, since many emodin-rich extracts deliver mixtures of free emodin and its glycosides, so the measured bioactivities may reflect in situ conversion to emodin and/or parallel activities of the conjugated species [8].

The highest and most consistently reported occurrence of emodin-related metabolites are found in species belonging to the families Polygonaceae and Fabaceae, which constitute the principal botanical sources of emodin used in both traditional medicine and modern phytochemical research [18,21]. Within Polygonaceae, species of the genus *Rheum* represent the most prominent and pharmacopoeially recognized sources. Medicinal rhubarb species, including *Rheum palmatum*, *R. officinale*, and *R. tanguticum*, accumulate emodin predominantly in their rhizomes and roots [8,22]. In these tissues, emodin is a characteristic anthraquinone marker and occurs alongside structurally related compounds such as rhein, chrysophanol, physcion, and aloe-emodin, forming the chemical basis of the biological activities attributed to rhubarb-derived preparations [23]. Because of this consistent presence and relatively high abundance, emodin is routinely employed as a quality-control and standardization marker for medicinal rhubarb preparations [24,25].

Closely related sources are found among species of *Polygonum* (syn. *Fallopia*), particularly *Polygonum cuspidatum* (Japanese knotweed) and *Polygonum multiflorum* (tuber fleeceflower) [20,26]. These plants are extensively used in traditional East Asian medicine and are chemically distinguished by the coexistence of emodin with other anthraquinones and additional classes of bioactive metabolites. In *P. cuspidatum*, emodin commonly co-occurs with stilbenes such as resveratrol, creating phytochemical profiles in which biological effects are likely to arise from multi-component synergistic interactions rather than from a single dominant constituent [26,27]. This diverse phytochemical profile is particularly relevant for extract-based studies, where synergistic or modulatory effects between anthraquinones and non-anthraquinone constituents may influence experimental outcomes.

Species of the genus *Cassia*, (e.g., *Cassia obtusifolia* and *Cassia tora*), commonly referred to as Cassiae semen in traditional medicine, represent another important botanical source of emodin. In these plants, emodin and related anthraquinones are concentrated mainly in the seeds and have been traditionally associated with laxative, digestive, and metabolic effects [28]. At the same time, the presence of emodin in *Cassia* species has prompted considerable attention from a toxicological perspective, as prolonged or high-dose exposure to anthraquinone-rich preparations has been linked to safety concerns [29]. As a result, *Cassia*-derived materials are frequently discussed in the context of both therapeutic potential and risk assessment.

Beyond medicinal plants, emodin is also encountered in alimentary contexts, most notably through the consumption of rhubarb as a food plant. While edible rhubarb stalks contain substantially lower levels of emodin than medicinal rhizomes and roots, they nevertheless represent a source of dietary exposure, particularly in populations where rhubarb is consumed regularly or in concentrated forms [30].

Interestingly, emodin-type anthraquinones are not confined to higher plants but also occur in several fungal taxa and lichens, which broadens both their ecological and pharmacognostic relevance [20,31]. Emodin and closely related congeners are produced by filamentous fungi, including species of *Aspergillus*, *Penicillium*, and other ascomycetes and basidiomycetes, and have been recognized as key intermediates in large fungal secondary-metabolite families such as xanthones, benzophenones, and grisandienes. Fungal fermentation, for example, can yield emodin at a preparative scale and is being explored as an alternative supply route to plant extraction; however, the toxicological profile of emodin-rich preparations from these sources will greatly differ from those of rhubarb or *Polygonum* roots due to the co-presence of unwanted polyketide metabolites (i.e., other anthraquinones and mycotoxins) [32].

## 4. Physicochemical and Pharmacokinetic Characteristics of Emodin

Emodin’s physicochemical properties are central to understanding its pharmacological behavior in biological systems and help explain frequent discrepancies observed between in vitro and in vivo efficacy. Its relatively low aqueous solubility, multiple ionizable phenolic groups, and moderate lipophilicity limit dissolution in gastrointestinal fluids and constrain passive absorption, while extensive phase II metabolism (notably glucuronidation) furthers systemic exposure after oral dosing [22,33]. As a result, high nominal concentrations readily achieved in cell cultures are often unattainable in plasma and tissues in vivo, making the direct extrapolation of in vitro efficacy or mechanistic insights to clinical context problematic. In addition, the need for relatively high oral doses to achieve therapeutic effects may increase the risk of dose-dependent toxicity, particularly in metabolically active organs such as the liver and kidneys [22].

Experimental and computational studies indicate that emodin is moderately lipophilic (logP in the 2–3 range) yet practically insoluble in water at neutral pH, leading to slow dissolution and dissolution-limited oral absorption. The presence of three phenolic OH groups confer pH-dependent ionization, making it poorly soluble and prone to aggregation in acidic and near-neutral aqueous solutions and much more soluble in alkaline media, where deprotonation and anionic forms are stabilized. From a pharmaceutical perspective, this implies that in the stomach and proximal small intestine, where absorption is typically most efficient, emodin predominantly exists in a neutral, poorly soluble form, which limits the fraction available for membrane permeation. In contrast, at the higher pH of the distal intestine, increased ionization enhances apparent solubility but may simultaneously reduce passive membrane permeability and promote more rapid clearance of the ionized species [20,34,35,36]. This pH-dependent balance between solubility and permeability therefore provides a clear rationale for salt formation and other formulation strategies aimed at improving overall bioavailability.

Low apparent absorption is not solely attributable to limited intestinal uptake but is strongly influenced by rapid intestinal and hepatic metabolism, which markedly reduces the fraction of parent compound reaching systemic circulation. Comprehensive pharmacokinetic profiling in rats indicates that the absolute oral bioavailability of emodin is approximately 2.8–3.2%, with roughly 50–60% of an orally administered dose remaining unabsorbed and eliminated in feces, reflecting inefficient gastrointestinal uptake and extensive presystemic metabolism [37,38]. Following absorption, emodin and its metabolites demonstrate broad but organ-selective tissue distribution. Rodent studies consistently report preferential distribution to the liver, kidneys, and gastrointestinal tissues, which correspond to the primary sites of metabolism, elimination, and reported toxicological effects. In contrast, penetration into the central nervous system appears limited under oral dosing conditions, suggesting that alternative formulation or delivery strategies may be required to achieve therapeutically relevant neuroprotective effects [39,40].

Importantly, tissue exposure does not directly correlate with plasma concentrations, as local accumulation (particularly in the liver) may occur despite low circulating levels of free emodin. Metabolism is a key determinant of emodin’s pharmacokinetic behavior and largely accounts for its low systemic exposure. While phase I pathways play a secondary role in overall clearance, phase II conjugation serves as the dominant metabolic pathway for emodin. In vivo studies indicate that rapid glucuronidation and sulfation, catalyzed by UDP-glucuronosyltransferases (UGTs) and sulfotransferases (SULTs), occurs extensively in both the intestinal mucosa and liver [41]. Nevertheless, it is notable that structurally related anthraquinones may also undergo interconversion via phase I oxidation pathways; specifically, chrysophanol (1,8-dihydroxy-3-methylanthraquinone) can be metabolically converted into aloe-emodin (1,8-dihydroxy-3-hydroxymethylanthraquinone) primarily through a cytochrome P450-dependent oxidation of the methyl side chain, further contributing to the dynamic anthraquinone metabolite pool in vivo [12].

Intestinal UGT activity, in particular, has been identified as a major contributor to presystemic metabolism, with substantial glucuronidation occurring prior to portal circulation entry. Consequently, emodin glucuronides and sulfates predominate in plasma, while the parent aglycone is often present only transiently or at trace concentrations following oral administration [37]. Plasma concentrations of unconjugated emodin typically decline rapidly after oral dosing and may become undetectable within a few hours, whereas conjugated metabolites persist at substantially higher concentrations. Although these metabolites are generally considered to possess reduced pharmacological activity, their sustained presence indicates ongoing systemic exposure to biotransformation products, which may contribute to tissue-specific effects [33,41]. Notably, anthraquinone glycosides have been reported to promote glucose uptake by inducing translocation of GLUT4 from intracellular light microsomal compartments to the plasma membrane in insulin-sensitive tissues [42], providing a mechanistic basis for the hypoglycemic effects observed with anthraquinone-rich preparations in vivo. 

In addition, the route of administration has a pronounced impact on emodin’s metabolic fate and the functional properties of its circulating metabolites. Following intravenous administration, parent emodin is rapidly cleared from the bloodstream, accompanied by the formation of emodin glucuronides as well as ω-hydroxyemodin and its sulfate and glucuronide conjugates. In contrast, oral administration may produce a distinct metabolite profile in which circulating metabolites may exhibit relatively greater free radical-scavenging capacity than those observed after intravenous dosing [43].

Interestingly, the bioavailability of emodin may be subject to more complex and context-dependent modulation depending on the specific composition of the preparation and the relative abundance of structurally related anthraquinones. Pharmacokinetic studies in rats show that the co-presence of related anthraquinone analogues in plant extracts leads to complex mutual pharmacokinetic modulation and overlapping pharmacodynamic profiles. In a comparative pharmacokinetic study of the five major rhubarb anthraquinones in rats, emodin, rhein, chrysophanol, physcion, and aloe-emodin altered each other’s Cmax, AUC, t½, clearance, and Vd, often in opposite directions, indicating competition or cooperation at the level of transporters, biotransformation enzymes, and tissue binding. For example, co-administration reduced emodin and rhein exposure while increasing aloe-emodin Cmax and prolonging its elimination, whereas physcion exerted particularly strong effects on aloe-emodin pharmacokinetics [44]. These reciprocal interactions are particularly important when interpreting studies based on plant extracts, as rhubarb, knotweed, and Cassia preparations typically contain complex mixtures of anthraquinones and their corresponding glycosides. Consequently, the observed biological effects often arise from the combined effects of closely related derivatives, rather than from the activity of an individual component. Furthermore, constituents within polyherbal preparations can substantially modulate the pharmacokinetics of anthraquinones, producing clinically relevant differences in exposure compared with single-herb products or isolated compounds. Multiple in vivo studies show that co-administered herbal components or concomitant drugs can increase or decrease the Cmax, AUC, and absorption rates of emodin, rhein, aloe-emodin, and chrysophanol by altering gastrointestinal motility, modifying transporter activity, or inhibiting phase II metabolism [20]. These findings demonstrate that formulation composition and herb–herb or herb–drug interactions are critical determinants of anthraquinone bioavailability and, ultimately, therapeutic outcomes. 

In view of the pharmacokinetic variability of the anthraquinone derivatives discussed throughout this section (low oral bioavailability, extensive phase II metabolism, transporter-mediated disposition, and interaction-driven modulation of systemic exposure), the key pharmacokinetic parameters reported for emodin across preclinical studies are summarized in Table 1. However, comparable quantitative PK datasets for the other anthraquinones are scarce, heterogeneous in terms of preparation (single compound vs. mixed extracts), and incomplete across major PK parameters, precluding a robust side-by-side comparison among these compounds.

## 5. Molecular and Cellular Mechanisms of Action

**Figure 4:**
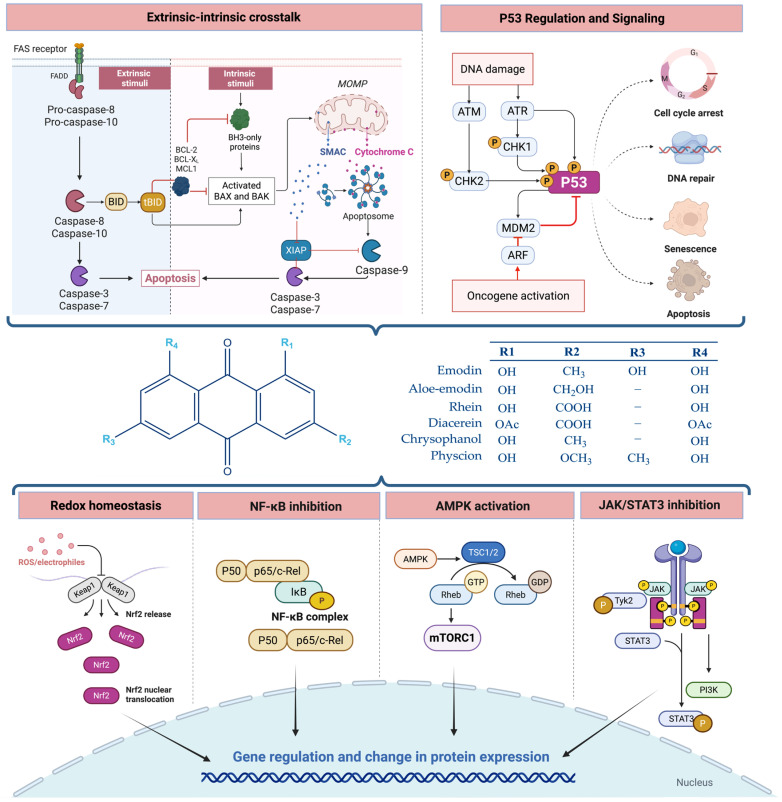
Apoptosis, inflammatory and metabolic signaling pathways targeted by anthraquinones: structural determinants and modulation of redox homeostasis, NF-κB, AMPK, and JAK/STAT3. Created in Biorender, Mihaylova R. (2026), https://BioRender.com/l29wxj6.

Molecular and cellular studies over the past two decades show that anthraquinone derivatives exert their broad biological effects by tuning interconnected signaling networks that govern inflammation, immunity, redox balance, metabolism, and cell fate. In this setting, emodin and its close analogues behave as context-dependent redox-active signaling modulators capable of reshaping inflammatory tone, reprogramming immune and stromal compartments, and redirecting tumor-cell survival or death. The following sections outline the major pathways and processes engaged by emodin-type anthraquinones, including oxidative stress and ferroptosis, NF-κB and JAK/STAT signaling, MAPK cascades, immune-cell differentiation, cell-cycle regulation, apoptosis, and metabolic and energy-sensing circuits, providing an integrated mechanistic framework for their anti-inflammatory, immunomodulatory, anticancer, and metabolic effects (Figure 4).

### 5.1. Modulation of Inflammatory Signaling

#### 5.1.1. Antioxidant vs. Pro-Oxidant Activity

Emodin is a redox-active anthraquinone whose biological effects reflect a context-dependent balance between antioxidant and pro-oxidant activity. At low to moderate concentrations, particularly in models of inflammatory or oxidative tissue injury, emodin exerts net antioxidant and cytoprotective effects by lowering ROS levels, limiting lipid peroxidation, and enhancing endogenous antioxidant defenses [49,50]. In a recent study of doxorubicin-induced cardiotoxicity, emodin significantly attenuated myocardial injury by suppressing ferroptosis, a regulated form of cell death driven by iron-dependent lipid peroxidation, supporting its role as an anti-oxidative and anti-ferroptotic modulator in this pathological setting [51]. In contrast, in cancer cells and other metabolically stressed conditions, the anthraquinone scaffold has been reported to act predominantly as a pro-oxidant, shifting redox homeostasis toward oxidative stress and promoting intracellular ROS accumulation, with reactive oxygen species functioning as active signaling mediators rather than passive by-products of cellular damage [52]. This dual behavior is characteristic of quinone-containing compounds and is shaped by dose, cellular redox status, metal (especially iron) availability, and pathological context. Anthraquinone moieties such as emodin can undergo one-electron reduction to semiquinone radicals, which subsequently redox-cycle with molecular oxygen to generate reactive oxygen species—a process that is amplified under conditions of iron abundance and weakened antioxidant defenses [52,53]. Emodin-induced ROS modulate classical stress and cell-death pathways, including MAPK cascades, p53 activation, and ferroptosis-related networks such as GPX4 suppression and lipid peroxidation. Moreover, emodin was shown to downregulate the expression of the multidrug resistance-associated protein 1 (MRP1) via a ROS-dependent mechanism, as this effect was reversed by antioxidant pretreatment with N-acetylcysteine (NAC) [53]. Other studies have likewise demonstrated that antioxidant exposure attenuates emodin-induced apoptosis, supporting a causal role for ROS as active mediators of its cytotoxic signaling [54,55,56]. Mechanistically, ROS can oxidize redox-sensitive cysteine residues in phosphatases and other regulatory proteins, biasing kinase activity, phosphorylation-dependent pathways, and transcriptional programs. In emodin-treated cells, ROS appear to act upstream of metabolic signaling changes, including ROS-dependent modulation of PI3K/AKT and GLUT1 expression, linking redox perturbation to metabolic and cell-fate responses [54]. In this context, ROS are best regarded as second messengers that couple emodin’s redox activity to downstream signaling pathways, rather than merely as non-specific damaging oxidants.

#### 5.1.2. Inhibition of Nuclear Factor-κB (NF-κB)

The nuclear factor-κB (NF-κB) signaling pathway is a central regulator of inflammatory responses, immune activation, cell survival, and proliferation. Dysregulated NF-κB activation contributes to the pathogenesis of chronic inflammatory diseases, cancer, metabolic disorders, and cardiovascular complications. 

Inhibition of nuclear factor-κB (NF-κB) signaling represents one of the most consistently reported molecular mechanisms underlying emodin’s anti-inflammatory activity. Across diverse cell types, including macrophages, epithelial cells, endothelial cells, and tumor cells, emodin suppresses NF-κB activation by inhibiting IκBα phosphorylation and subsequent degradation, thereby preventing the nuclear translocation of the p65/p50 heterodimer and limiting the transcription of pro-inflammatory target genes [22]. At the upstream level, emodin interferes with Toll-like receptor 4 (TLR4)/MyD88-dependent signaling, resulting in reduced activation of the IκB kinase (IKK) complex and attenuation of downstream NF-κB signaling. This mechanism has been validated in multiple in vivo models of inflammatory disease, including experimental colitis, acute lung injury, and atherosclerosis, where suppression of NF-κB signaling is associated with a decreased expression of key inflammatory mediators such as TNF-α, IL-1β, IL-6, COX-2, and iNOS, as well as reduced inflammatory cell infiltration into affected tissues [22,57,58].

Mechanistic studies further indicate that emodin modulates innate immune activation by targeting TLR-mediated pathways, particularly the TLR4/MyD88 axis, which plays a central role in initiating inflammatory responses. Inhibition of TLR4-dependent adaptor recruitment and downstream kinase activation dampens signal propagation toward the IKK–NF-κB cascade, thereby limiting excessive inflammatory signaling. This mode of action aligns with broader immunomodulatory strategies aimed at restoring immune homeostasis and restraining pathological inflammation, as emphasized in the contemporary immunology literature [59].

#### 5.1.3. Inhibition of JAK/STAT Signaling

The Janus kinase/signal transducer and activator of transcription (JAK/STAT) pathway is a central regulator of cytokine-driven immune responses, inflammation, cell proliferation, and survival. Aberrant activation of the JAK/STAT signaling axis is a hallmark of chronic inflammatory, autoimmune, and malignant diseases, making this pathway an attractive pharmacological target [60]. Accumulating evidence across diverse experimental models indicates that emodin-related anthraquinones negatively regulate JAK/STAT signaling by reducing the phosphorylation of STAT3 and STAT1, key transcription factors downstream of JAK kinases—a mechanism that strongly contributes to their anti-inflammatory and immunomodulatory profile. In hepatocellular carcinoma cell lines (HepG2, Huh-7), emodin decreases both constitutive and interleukin-6 (IL-6)-induced STAT3 activation in a dose- and time-dependent manner by inhibiting upstream JAK1/JAK2 and c-Src activity and by upregulating the tyrosine phosphatase SHP-1 [61]. Notably, emodin has also been shown to modulate JAK/STAT signaling in a manner that intersects with interferon pathways and tumor immune surveillance. In both leukemia and solid tumor-cell systems, emodin suppressed aberrant STAT3 activation, in part through inhibition of casein kinase 2 (CK2) and JAK2, leading to a reduced expression of pro-survival and proliferative genes. In contrast, in cervical carcinoma (HeLa) and hepatocellular carcinoma (Huh-7) cells, the anthraquinone enhanced type I interferon (IFN-α/β) signaling by promoting STAT1 phosphorylation while concomitantly attenuating STAT3 activation, shifting the balance toward a pro-apoptotic interferon-driven transcriptional program. Consistent with these in vitro findings, combined treatment with emodin and IFN-α in a Huh-7 xenograft model resulted in enhanced intratumoral STAT1 phosphorylation, reduced STAT3 signaling, stabilization of the interferon receptor IFNAR1, and significantly greater inhibition of tumor growth compared with IFN-α monotherapy [62]. These data support a dual, context-dependent role of emodin in JAK/STAT signaling, characterized by the inhibition of aberrant STAT3 activity in inflammatory and neoplastic settings and the selective amplification of STAT1-mediated antitumor immunity under IFN-α exposure.

In a murine model of sepsis-induced jejunal injury, emodin attenuated intestinal inflammation and barrier damage through modulation of JAK1/STAT3 signaling in the gut, indicating that its influence on this pathway is not restricted to tumor settings but extends to systemic inflammatory stress [63]. More recently, in a cystitis model, emodin inhibited mast cell migration in vitro and in vivo by downregulating JAK2/STAT3 phosphorylation and suppressing the JMJD3/CXCR3 axis, thereby preventing the chemokine-driven accumulation of mast cells in inflamed bladder tissue [64]. In macrophages, emodin has been reported to bidirectionally modulate polarization by inhibiting NF-κB/IRF5/STAT1 and IRF4/STAT6 signaling pathways, resulting in reduced transcription of both M1- and M2-associated gene programs and promoting the restoration of macrophage homeostasis under inflammatory conditions [65]. 

Aloe-emodin and other structurally related anthraquinones appear to engage overlapping JAK/STAT-associated mechanisms, frequently in concert with additional signaling nodes. For example, in neuroblastoma and glioma models, aloe-emodin displays pronounced antitumor activity linked to the modulation of ERK1/2 and other growth factor-driven pathways [66]. Recent mechanistic reviews further indicate that anthraquinones such as aloe-emodin, rhein, chrysophanol, and their glycosides are able to intersect with JAK/STAT signaling as part of a broader regulatory network that includes PI3K/Akt, MAPK, and NF-κB pathways in both cancer and inflammatory contexts [67,68,69]. For example, the anthraquinone glycosides pulmatin (chrysophanol-8–0-glucoside) and emodin-8-glucoside have been reported to possess prominent JAK2 inhibitory activity, surpassing that of flavonoids, chlorogenic acid, and other widely explored polyphenols [70].

#### 5.1.4. Modulation of the MAPK Signaling Pathways

The mitogen-activated protein kinase (MAPK) signaling pathways, including extracellular signal-regulated kinase (ERK1/2), c-Jun N-terminal kinase (JNK), and p38 MAPK, play critical roles in regulating cell proliferation, differentiation, stress responses, inflammation, and apoptosis. Dysregulation of these pathways is implicated in chronic inflammatory diseases, cancer, and metabolic disorders. Most of the discussed anthraquinones have been reported to exert inhibitory effects on the MAPK/ERK signaling pathway; however, results for certain representatives, such as aloe-emodin, emodin, and chrysophanol, are inconsistent across studies, with reports of both inhibition and transient activation [22,71]. Importantly, such transient MAPK activation may reflect a cellular stress response rather than a proliferative signal, frequently preceding growth arrest or apoptosis. Nevertheless, the majority of experimental studies indicate that emodin suppresses activation of the stress-responsive MAPKs JNK and p38, particularly under inflammatory or oxidative stress conditions. In both cellular and animal LPS-sensitized models, emodin significantly reduces the phosphorylation of JNK and p38, leading to attenuation of downstream transcription factors such as AP-1 and reduced expression of pro-inflammatory genes. This inhibitory effect on JNK/p38 signaling correlates with the decreased production of inflammatory mediators, including TNF-α, IL-6, IL-1β, COX-2, and iNOS, supporting a central role for MAPK suppression in emodin’s anti-inflammatory profile [22,43]. While suppression of JNK/p38 predominates in inflammatory settings, in certain oncologic contexts, as in KLE endometrial carcinoma cells, emodin elicited a distinct signaling response characterized by transient activation of MAPK components. In this model, emodin induced the phosphorylation of p38, ERK, and JNK while concomitantly suppressing AKT phosphorylation in a dose- and time-dependent manner. Rather than promoting proliferation, this pattern of MAPK activation is associated with cell-cycle arrest and apoptotic signaling, reflecting the activation of stress-induced, pro-death pathways [72]. This dual modulation highlights both the pleiotropic nature of ERK signaling and emodin’s function as a context-dependent modulator of MAPK pathways rather than a nonspecific MAPK inhibitor [73].

#### 5.1.5. Immunomodulatory Activity

Accumulating evidence positions anthraquinones as a chemically tractable platform for tuning adaptive and innate immune responses through coordinated control of T-cell subsets, DC maturation, and IL 1/NLRP3 centered cytokine circuits [67,74]. Within T-cell populations, emodin modulates the balance among CD4^+^ helper subsets. In vitro and in vivo studies consistently show the suppression of Th1- and Th17-associated inflammatory outputs, including interferon-γ (IFN-γ), interleukin-2 (IL-2), and IL-17, alongside attenuation of excessive Th2 responses, recalibrating both cell-mediated and antibody-dependent adaptive immune responses [67]. It also reduces the frequency of IFN-γ^+^ and IL-4^+^ CD4^+^ and CD8^+^ T cells in peripheral blood mononuclear cells and splenic lymphocytes, supporting an anti-inflammatory cytokine milieu [75]. In severe inflammatory settings, including acute pancreatitis and systemic inflammation models, emodin restores Th1/Th2/Th17 equilibrium and suppresses IL-17 and IFN-γ production by γδ T cells, further highlighting its capacity to normalize dysregulated T-cell responses [76].

A qualitative remodeling has also been reported for the regulatory T-cell populations. In human PBMC-derived systems, emodin increased CD4^+^CD25^+^ Treg counts but yielded Tregs with reduced expression of HLA-DR, GITR, and CTLA-4, indicating the regulation of both Treg abundance as well as phenotype [77]. In a stringent murine skin allograft model, the anthraquinone prolonged graft survival, expanded CD4^+^FoxP3^+^ and CD8^+^CD122^+^ Treg populations, enhanced their suppressive function, and concomitantly reduced effector CD8^+^ T-cells and alloantibody production [74]. Functionally, these changes correlate with enhanced suppressive capacity and improved tolerance in transplantation and inflammatory disease models.

Dendritic cells (DCs) represent another target axis for anthraquinones’ immunomodulatory activity. In human monocyte-derived DCs, emodin inhibits their differentiation and maturation, downregulates the surface expression of CD80 and CD83, reduces IL-12p70 secretion, and halves the capacity of DCs to drive allogeneic T-cell proliferation while increasing anti-inflammatory IL-10 production [77]. In vivo, emodin similarly hinders DC maturation after transplantation, lowering CD11c^+^CD80^+^/CD86^+^ subsets in lymphoid tissues and contributing to reduced T-cell priming [74]. Given the established role of CD83 in maintaining DC immunogenic competence and tolerance balance, emodin-mediated CD83 suppression provides a mechanistic basis for reduced signal-2 delivery at the APC–T-cell interface [78,79]. Interestingly, other anthraquinones can have opposite effects: physcion enhances DC maturation; upregulates CD40/CD80/CD86/MHC II; increases IL-12p70, IL-1β, IL-6, and TNF-α; and selectively promotes Th1 polarization, pointing to scaffold-dependent preferences in immune modulation [67].

Related anthraquinones have also demonstrated interesting patterns of immune regulation. For rhein and its prodrug diacerein, the best-characterized immunomodulatory axis centers on IL-1 signaling. Diacerein and rhein inhibit IL-1β synthesis, reduce IL-1 receptor expression on chondrocytes and synoviocytes, and blunt downstream NF-κB/MAPK activation, thereby decreasing TNF-α, IL-6, and MMP production in osteoarthritic tissues [80], although fewer studies have dissected their effects on T-cell subsets in detail. More recently, both diacerein and its metabolite rhein have been shown to suppress NLRP3 inflammasome activation and IL-1β release in SARS-CoV-2-infected PBMCs, linking anthraquinone scaffolds to inflammasome-directed immunoregulation in viral infection diseases [81].

### 5.2. Regulation of Cell Proliferation and Apoptosis

Emodin and closely related anthraquinones are reported to regulate tumor-cell fate through the coordinated regulation of cell-cycle progression and apoptotic signaling. In many cancer models, growth inhibition is initiated by cell-cycle arrest, which frequently precedes and facilitates the activation of irreversible cell-death pathways [82,83,84].

#### 5.2.1. Cell-Cycle Arrest and Growth Inhibition

Emodin inhibits cell proliferation by enforcing cell-cycle arrest, most commonly at the G0/G1 or G2/M checkpoints, depending on cell type, exposure conditions, and molecular context. This effect is mechanistically linked to the downregulation of key cyclins (including cyclin D1 and cyclin B1) and cyclin-dependent kinases (CDK2, CDK4), alongside the upregulation of CDK inhibitors, particularly p21^Cip1/Waf1. Through these coordinated changes, emodin disrupts orderly cell-cycle progression and imposes a cytostatic state that primes cells to subsequent apoptotic execution [82,83]. Notably, the enforcement of a defined cell-cycle arrest state may synchronize tumor-cell populations and thereby enhance susceptibility to apoptosis or sensitize cells to combination treatments, suggesting a potential basis for synergistic interactions with cell-cycle-dependent therapies, such as DNA-targeting chemotherapeutics, antimitotic agents, or radiotherapy. For example, Trybus et. al. demonstrated that emodin synergistically potentiates vinblastine-induced cytotoxicity by enforcing G2/M arrest, amplifying ROS-driven mitotic catastrophe, mitochondrial damage, and caspase-3/7-dependent apoptosis [85]. Similar results were also achieved against gastric cancer cells, where emodin markedly enhanced cisplatin responsiveness and proapoptotic signaling [86].

This growth-suppressive activity is shared by structurally related anthraquinones, although the precise checkpoint targeted may vary. For example, emodin and its analogs consistently suppress tumor-cell proliferation by enforcing cell-cycle checkpoint arrest, most commonly at G0/G1 or G2/M, with the exact phase depending on cell type, compound, and exposure conditions [9,82,87,88]. In human colon cancer (WiDr) cells, aloe-emodin induced a pronounced G2/M arrest associated with suppression of cyclin B1, a critical regulator of mitotic entry. In this model, cell-cycle blockade was tightly coupled to caspase-6-dependent apoptosis execution, linking checkpoint arrest to downstream cell-death pathways in response to anthraquinone exposure [82].

#### 5.2.2. Activation of the Mitochondrial (Intrinsic) Apoptotic Pathway

Downstream of this growth arrest, the mitochondrial (intrinsic) pathway emerges as the dominant route of emodin-induced apoptosis across multiple malignancies, including hepatocellular, pancreatic, gastric, and colorectal cancer models. Emodin disrupts mitochondrial membrane potential—an early and decisive event in intrinsic apoptosis—leading to loss of mitochondrial integrity and the release of pro-apoptotic factors into the cytosol [89]. A key mechanistic feature of emodin in this context is its ability to shift the balance of Bcl-2 family proteins toward a pro-apoptotic phenotype, characterized by the upregulation of Bax and Bak alongside the downregulation of anti-apoptotic members such as Bcl-2 and Bcl-xL. As a result, mitochondrial outer-membrane permeabilization (MOMP) is promoted, enabling cytochrome c release and subsequent apoptosome formation through association with Apaf-1. Activation of initiator caspase-9 follows, triggering executioner caspases, notably caspase-3 and caspase-7, and culminating in apoptotic cell death [90].

Importantly, mitochondrial apoptosis induced by emodin appears to be tightly linked to oxidative stress signaling. The emodin-driven generation of reactive oxygen species (ROS) contributes to mitochondrial dysfunction, the modulation of Bcl-2 family proteins, and caspase activation. The pharmacological attenuation of ROS has been shown to partially reverse emodin-induced apoptosis, supporting the central role of ROS in mitochondrial permeabilization and anthraquinone-induced cell death [90].

#### 5.2.3. Death Receptor Signaling and Extrinsic–Intrinsic Crosstalk

In parallel, emodin can engage components of the extrinsic, death-receptor-mediated apoptotic pathway. Activation of caspase-8 and sensitization to Fas/FasL or TRAIL signaling have been reported, particularly in combination settings where emodin potentiates chemotherapy- or cytokine-induced apoptosis [91,92,93]. Importantly, extrinsic–intrinsic apoptotic crosstalk is most frequently observed in combinatorial or sensitized scenarios, rather than as a universal response to emodin alone. In human liver cancer cells, emodin has been reported to activate caspase-8 together with mitochondrial apoptosis markers (including cytochrome c release and caspase-9 activation), indicating the coordinated recruitment of both death-receptor and intrinsic cascades [91]. These apoptotic events occurred alongside the cleavage of Bid to tBid, which amplifies death receptor signaling through mitochondrial permeabilization (extrinsic–intrinsic crosstalk) [94]. In addition, emodin frequently acts as an apoptosis sensitizer in combination regimes: by facilitating DISC formation and caspase-8 activation, it enhances death-receptor-driven apoptosis induced by chemotherapeutic agents, leading to stronger caspase-3/-7 activation and reduced clonogenic survival, as documented for hepatocellular and colorectal cancer models [91,93,95].

#### 5.2.4. Modulation of p53

The tumor suppressor p53 represents an important, albeit context-dependent, molecular target in emodin’s regulation of cell proliferation and apoptosis. In p53-competent experimental models, emodin was found to stabilize p53 and enhance the transcription of pro-apoptotic target genes, integrating ROS and stress-activated kinase signaling (notably p38 MAPK) with mitochondrial apoptosis. Recent reviews highlight the involvement of the ROS-p38-p53-PUMA axis in colorectal cancer models, where emodin promotes apoptosis through coordinated redox and stress signaling [93]. Notably, aloe-emodin retains the ability to induce caspase-8-mediated apoptosis in p53-deficient or p53-mutant cells, indicating the existence of p53-independent mechanisms that broaden the applicability of anthraquinones across genetically diverse tumors [96,97,98]. However, current evidence for anthraquinone-mediated p53 modulation in primary human cells is largely confined to preclinical studies and requires validation in clinical settings. Beyond oncology, p53-mediated growth arrest and apoptosis have also been documented in vascular smooth muscle cells, where emodin-induced ROS accumulation and p53 activation contribute to the inhibition of pathological proliferation relevant to restenosis and atherosclerotic remodeling [38].

### 5.3. Modulation of Metabolic and Energy-Sensing Pathways

Beyond their direct effects on cell proliferation and inflammatory signaling, emodin-type anthraquinones exert broad regulatory actions on cellular metabolism by targeting energy-sensing and immunometabolic pathways. Several representatives, most notably rhein and diacerein, but also aloe-emodin, chrysophanol, and physcion, have been reported to exert multimodal effects on glucose and lipid metabolism, insulin sensitivity, and obesity-related traits. A recurring theme across diverse experimental models is the activation of AMP-activated protein kinase (AMPK)—a master regulator that integrates energetic stress, redox status, and inflammatory signals [7,99]. Complementary AMPK-independent mechanisms also contribute to the improvement of metabolic homeostasis by emodin and related anthraquinone derivatives in disease-relevant models.

Emodin itself demonstrates broad, but so far primarily preclinical, metabolic benefits that are mechanistically driven by AMPK activation and coordinated immunometabolic reprogramming. A systematic review and meta-analysis of T2DM animal models showed that emodin consistently improved fasting blood glucose, glucose tolerance, and insulin sensitivity while lowering total cholesterol, triglycerides and LDL-cholesterol and partially normalizing HDL-cholesterol, across multiple dosing regimens and models [99,100]. Mechanistic studies consolidated in a recent review on anthraquinones and insulin resistance indicate that emodin activates AMPK in the liver and skeletal muscle of high-fat-diet rodents, increases CPT-1 expression, suppresses SREBP-1c and fatty acid synthase, enhances fatty acid oxidation, and reduces hepatic lipid accumulation, thereby improving insulin sensitivity and systemic metabolic homeostasis [101]. In addition, emodin has also been identified as a potent and selective inhibitor of 11β-hydroxysteroid dehydrogenase type 1 (11β-HSD1)—a key enzyme linking glucocorticoid excess to insulin resistance and metabolic syndrome. In biochemical in vitro and in vivo studies, emodin inhibited human and murine 11β-HSD1 at nanomolar potency, reduced hepatic and adipose enzyme activity after oral administration, and selectively reversed prednisone-induced insulin resistance, confirming target engagement in vivo. In diet-induced obese mice, emodin improved insulin sensitivity, normalized lipid metabolism, lowered blood glucose, and suppressed hepatic gluconeogenic gene expression, including PEPCK and glucose-6-phosphatase [102]. These findings place emodin as a broadly effective metabolic modulator in vivo, although its clinical evaluation falls behind that of other anthraquinones in the family, such as diacerein [103,104,105,106,107].

## 6. Therapeutic Potential in Disease Models

The anthraquinone scaffold has emerged as a versatile pharmacophore with well-documented activities across malignant, inflammatory, metabolic, cardiovascular, and neurodegenerative disease settings, although the strength and maturity of evidence vary by compound and indication. Within the family, emodin is the most intensively studied representative, showing multi-model in vitro and in vivo efficacy, while other congeners, notably diacerein, achieve stronger clinical validation in selected niches such as diabetic kidney disease and chronic joint disorders. The following subsections synthesize this disease-oriented evidence base, with particular emphasis on emodin’s profile and how it compares to its analogues across key domains of redox control, inflammation, metabolism, and regulated cell death. Furthermore, to ensure a transparent and structured comparison of evidence strength across compounds and therapeutic domains, a systematic literature mapping approach was applied, integrating quantitative evidence stratification and a semi-quantitative scoring framework. This methodology enabled consistent evaluation of translational maturity from mechanistic in vitro studies through in vivo validation and, where available, clinical investigation.

### 6.1. Methodology

#### 6.1.1. Literature Search Strategy

A systematic literature search was conducted in PubMed/MEDLINE to identify primary research studies investigating the biological activities of major anthraquinone derivatives (emodin, aloe-emodin, rhein, chrysophanol, physcion, catenarin, and diacerein). The search covered the period from January 2000 to February 2026. For each compound, domain-specific searches were performed using predefined keyword combinations related to five biological domains: antioxidant, anti-inflammatory, neuroprotective, anticancer, and metabolic disorders. Representative search strategies included combinations such as

- (“emodin” AND “antioxidant” AND (“in vitro” OR “in vivo”)).
- (“rhein” AND “anti-inflammatory”).
- (“chrysophanol” AND “neuroprotective”).
- (“physcion” AND (“metabolic syndrome” OR “diabetes” OR “dyslipidemia”)).

Search strings were adapted to each compound–domain pair. Only primary research articles were included. Reviews, meta-analyses, editorials, conference abstracts without full primary data, duplicate records, and non-relevant studies were excluded.

#### 6.1.2. Study Screening and Evidence Stratification

All retrieved records were screened for relevance based on title and abstract, followed by full-text assessment where necessary. Studies were included if they investigated the respective anthraquinone within one of the predefined biological domains and reported primary experimental or clinical data.

Eligible studies were categorized into three evidence tiers: in vitro (cell-based, biochemical, or mechanistic assays), in vivo (animal experimental models), and clinical (human interventional or observational studies). For each compound–domain pair, the number of included studies was calculated as

Included studies = Identified records − Excluded records

Excluded records comprised non-relevant studies (e.g., wrong compound, unrelated outcome, inappropriate model), duplicates, and non-primary publications.

#### 6.1.3. Semi-Quantitative Evidence Scoring Framework

To comparatively evaluate the strength and translational maturity of the available evidence, a structured semi-quantitative scoring system (0–4) was applied. Scores were assigned hierarchically according to the highest level of validated evidence and predefined numerical thresholds:

Score 4: Reflecting direct translational validation in humans, assigned when the following criterion was met:

- More than one eligible clinical study was identified, irrespective of preclinical evidence volume.

Score 3: Reflecting strong preclinical evidence, assigned when both of the following criteria were met:

- Included in vitro studies ≥50 and included in vivo studies ≥50.

Score 2: Reflecting moderate preclinical evidence but consistent mechanistic findings across in vitro and in vivo models, assigned when either of the following criteria were met:

- Included in vitro studies ≥20 and included in vivo studies = 10–49, or included in vitro studies ≥50 with included in vivo studies <10.

Score 1: Limited or emerging evidence, assigned when either of the following criteria were met:

- Included in vitro studies = 1–19 with included in vivo studies = 0, or included in vivo studies = 1–9, regardless of in vitro volume.

Score 0: No convincing evidence, assigned when the following criteria were met:

- Included in vitro studies = 0 and included in vivo studies = 0.

#### 6.1.4. Normalization and Comparative Visualization

**Figure 5:**
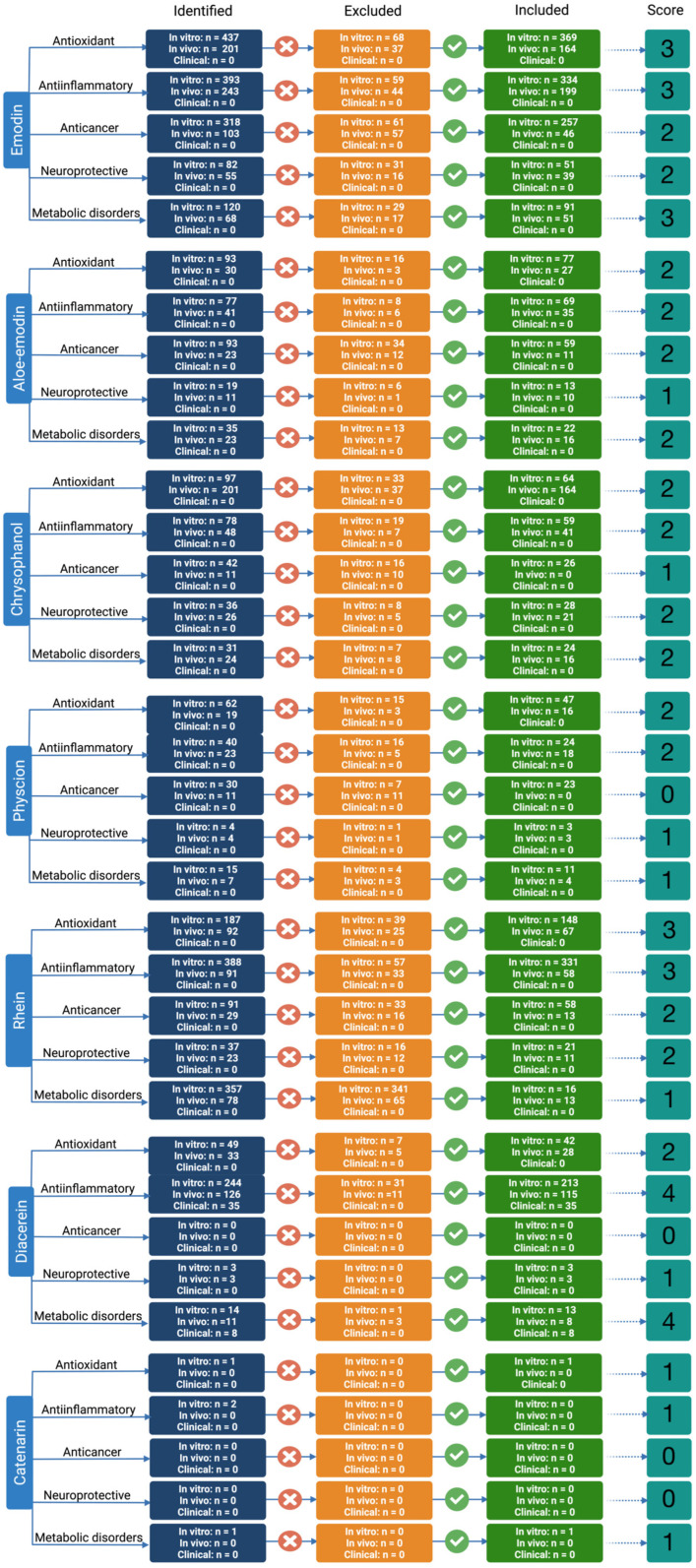
Evidence flowchart and strength-of-evidence scoring for anthraquinone derivatives across major biological domains. Created in Biorender, Mihaylova R. (2026), https://BioRender.com/vwc3r9k.

**Figure 6:**
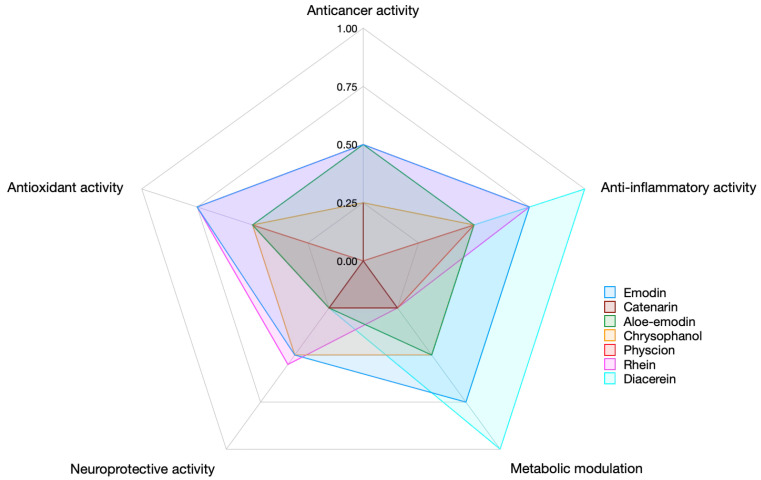
Comparative radar plot showing the relative evidence strength for emodin and related anthraquinone derivatives across major biological domains, including anticancer, anti-inflammatory, metabolic, neuroprotective, and antioxidant activities. Scores (0–4) (Figure 5), normalized to 0–1 for visualization, reflect integrated preclinical and clinical evidence as described in Section 6.1 and depict relative activity trends rather than absolute pharmacological potency.

For comparative graphical representation across compounds and domains, raw scores were normalized as: normalized score = Score/4, yielding values ranging from 0.00 to 1.00, that reflect relative evidence strength and translational potential (Figure 5 and Figure 6).

#### 6.1.5. Limitations of the Evidence Mapping and Scoring Approach

Several methodological limitations should be considered when interpreting the evidence mapping and semi-quantitative scoring results. First, partial overlap between in vitro and in vivo datasets is inherent to preclinical pharmacological research, as many animal studies are preceded or accompanied by mechanistic cellular experiments. Although studies were counted separately by evidence tier to reflect translational progression, this may lead to partial redundancy in mechanistic evidence representation. Second, biological domains included in the analysis are not fully independent. Key molecular pathways, such as oxidative stress modulation, inflammatory signaling, mitochondrial dysfunction, and regulated cell death, frequently intersect across antioxidant, anti-inflammatory, neuroprotective, anticancer, and metabolic outcomes. Consequently, individual studies may contribute mechanistic insight to multiple domains, potentially inflating apparent evidence breadth despite reflecting shared upstream processes. Third, heterogeneity in experimental design, disease models, dosing regimens, and outcome measures across studies was not formally weighted within the scoring framework. The semi-quantitative approach prioritized evidence volume and tier progression rather than effect size or methodological quality, which may oversimplify differences in robustness between individual investigations. Fourth, part of the available literature, particularly in the metabolic and anticancer domains, involves complex botanical extracts or fractions enriched in anthraquinones rather than isolated pure compounds. Although such studies were included when the anthraquinone contribution was clearly stated, the presence of multiple bioactive constituents may confound the attribution of observed effects to a single compound. Finally, reliance on PubMed/MEDLINE as the primary data source may have excluded relevant studies indexed exclusively in other databases or regional journals. While the chosen databases capture the majority of the biomedical literature, minor publication bias cannot be excluded. Moreover, while a systematic compound-by-compound evaluation of all reported biological activities would have been ideal to fully capture the therapeutic breadth of the anthraquinone scaffold, such an exhaustive analysis, this would have exceeded the intended scope of the present review. Therefore, the present subsection is best interpreted as a structured evidence-mapping review with semi-quantitative synthesis rather than a formal PRISMA-compliant systematic work.

### 6.2. Therapeutic Potential in Malignant Diseases

Within the oncological landscape, emodin has emerged as a particularly versatile anthraquinone, with reproducible antitumor efficacy demonstrated in multiple in vitro and vivo models, especially across solid carcinomas including lung, liver, colon, breast, pancreatic, cervical, and ovarian cancers [9]. Accumulating evidence indicates that emodin targets not only tumor cells directly but also modulates the tumor immune microenvironment and, more recently, it has been shown to engage ferroptotic cell-death pathways [57,84]. While other anthraquinones such as aloe-emodin, rhein, and diacerein also exhibit in vivo anticancer activity, their supporting evidence is generally more indication-specific and less extensive than that currently available for emodin.

Accumulating data from gastrointestinal models demonstrate that emodin’s anticancer potential is particularly well supported in solid tumors, with colorectal cancer (CRC) being the best-characterized indication. In murine sporadic CRC models, including both genetically engineered strains and azoxymethane/dextran sulfate sodium-induced colitis-associated cancer, emodin significantly reduced tumor number and size while dampening tumor-associated inflammation. These antitumor effects were accompanied by a marked decline in M2-polarized macrophage infiltration in the colonic tumor microenvironment, indicating that emodin exerts both tumor cell-intrinsic effects and immune-microenvironment reprogramming, in line with current concepts of inflammation-driven colorectal tumorigenesis [108]. In xenograft-based CRC models, emodin inhibited tumor growth by activating ferroptosis-related pathways; mechanistic studies found increased lipid peroxidation, downregulation of GPX4, activation of NCOA4-mediated ferritinophagy, and inhibition of NF-κB signaling both in vitro and in vivo, establishing ferroptosis as a bona fide contributor to emodin’s antitumor activity rather than a purely in vitro phenomenon [57].

Moreover, emodin’s efficacy extends beyond the gastrointestinal tract to other solid tumor types, particularly bladder cancer, where it exerts pronounced effects on the tumor microenvironment. In a murine bladder cancer model, emodin significantly inhibited tumor growth by reducing tumor-associated macrophage (TAM) accumulation—an effect mechanistically linked to selective suppression of CXCL1 secretion by cancer-associated fibroblasts (CAFs). Disruption of this CAF-TAM axis impaired TAM migration and was essential for emodin’s antitumor activity, as tumor growth inhibition was lost when CXCL1-deficient CAFs were present [109].

By contrast, in vivo evidence for emodin in hematological malignancies is more limited and points toward a role as a chemosensitizer rather than a standalone cytotoxic agent. Emodin enhanced cytarabine (Ara-C) chemosensitivity in resistant HL-60/ADR cells in vitro as well as in an acute myeloid leukemia (AML) xenograft model, where it reduced leukemic burden and improved survival via the inhibition of Akt and ERK signaling [110]. Emodin derivatives such as E35 have been developed to improve in vivo efficacy in AML models, demonstrating enhanced antiproliferative activity compared to the parent compound. Notably, the pharmacological inhibition of autophagy with 3-methyladenine markedly potentiated E35-induced apoptosis in AML cell lines, patient-derived cells, and xenograft models, resulting in enhanced leukemia growth inhibition without overt toxicity [111].

Within the wider anthraquinone family, several congeners also show meaningful anticancer activity in vivo, though the depth and breadth of evidence vary. Aloe-emodin has been investigated most extensively after emodin, particularly in prostate and lung cancer models. 

Phosphatidylinositol 3-kinase (PI3K) amplification and loss of the tumor suppressor phosphatase and tensin homolog (PTEN) are key oncogenic events that drive aberrant Akt activation in prostate cancer. In a PTEN-deficient xenograft mouse model of prostate cancer, aloe-emodin suppressed tumor-cell survival and growth by inhibiting mTORC2 activity and interfering with the downstream PI3K/Akt axis signaling [112].

A comprehensive systematic review by Sander et. al. indicates that aloe-emodin exhibits broad antiproliferative and anti-carcinogenic activity across diverse human cancer cell lines, often through the simultaneous modulation of multiple signaling pathways. Reported effects include inhibition of proliferation, migration, and invasion; induction of cell-cycle arrest and programmed cell death; disruption of mitochondrial function and redox balance; and regulation of immune-related signaling. Importantly, these activities are highly context-dependent, with particularly pronounced effects documented in lung cancer subtypes, as well as in human nasopharyngeal carcinoma, tongue squamous cell carcinoma, and melanoma models [98]. More recently, aloe-emodin-based derivatives such as its 3-O-glucoside have shown potent growth-inhibitory and pro-apoptotic effects in non-small-cell lung cancer xenografts by suppressing MEK/ERK and Akt pathways, pointing to a route for improving pharmacokinetics and tumor selectivity [113].

Rhein also displays notable antitumor activity, particularly in breast cancer models, where both the parent compound and optimized formulations have been tested. In triple-negative breast cancer, a rhein derivative (4F) inhibits RAC1 signaling and suppresses the proliferation, migration, and invasion of MDA-MB-231 cells, while hyaluronic-acid-modified liposomal rhein enhances tumor targeting and significantly improves antitumor efficacy in 4T1 breast tumor-bearing mice [114].

Diacerein, primarily developed as an anti-inflammatory and antidiabetic agent, has recently been repurposed in oncology: in a murine Ehrlich solid tumor model, diacerein combined with 5-fluorouracil significantly reduced tumor weight and volume, prolonged survival, decreased oxidative stress, inhibited AKT phosphorylation and NF-κB-driven inflammation, and enhanced apoptosis via modulation of Bax, Bcl-2, p53, and caspase-3 [115]. Diacerein has also been shown to improve the penetration and therapeutic efficacy of radiolabeled rituximab in a Burkitt lymphoma xenograft model, suggesting a broader chemosensitizing and “microenvironment-modifying” role across solid and hematologic tumors [116].

### 6.3. Therapeutic Potential in Inflammatory and Autoimmune Diseases

Inflammatory and autoimmune diseases are driven by tightly interlinked networks of oxidative stress, cytokine signaling, and immune dysregulation, sustaining pathological inflammation and chronic tissue damage. Many of the natural anthraquinones and their semi-synthetic derivatives (e.g., diacerein), have been reported to act as multi-target modulators of these processes both in vitro and in vivo. Within the plant-derived anthraquinone class, the most robust preclinical evidence currently supports emodin in models of intestinal, joint, and lung inflammation, whereas the diacetyl prodrug of rhein diacerein stands out for its clinical efficacy in rheumatic disease and osteoarthritis. Other anthraquinone congeners, such as chrysophanol and physcion, contribute additional anti-inflammatory activity in metabolic and renal inflammatory contexts; however, their roles in models of classical autoimmune diseases are less comprehensively characterized than those of emodin and diacerein in the current literature base.

Emodin shows reproducible efficacy across multiple murine models of colitis, with a particularly strong evidence base in DSS-induced ulcerative colitis and AOM/DSS colitis-associated cancer. In the AOM/DSS model, oral emodin (50 mg/kg) reduced the incidence, number, and size of colonic neoplasms while attenuating colitis severity, decreasing inflammatory cell infiltration and lowering mucosal expression of pro-inflammatory cytokines and NF-κB-regulated mediators, indicating the simultaneous suppression of inflammation and inflammation-driven tumorigenesis. At earlier time points, emodin shifted lesion histology from high-grade dysplasia/carcinoma toward low-grade adenomas and reduced the recruitment of myeloid cells and granulocytic MDSCs, further underscoring its ability to remodel the intestinal inflammatory microenvironment in vivo [117]. In acute DSS colitis, free emodin improved body-weight loss, disease activity index, colon shortening, and histological injury, but its poor solubility and rapid upper-GI metabolism have driven an intensive wave of formulation-oriented approaches [118]. Colon-targeted nanoparticles based on PLGA (Poly(DL-lactide-co-glycolide)), designed to prevent premature release in the stomach and small intestine, selectively accumulate emodin in inflamed colonic mucosa and significantly outperform the free form in reducing myeloperoxidase activity, fecal lipocalin-2, and barrier disruption [118].

In pulmonary inflammation, emodin again shows consistent protective effects in acute lung injury (ALI) and related settings. In an LPS-induced ALI rat model, emodin significantly reduced pulmonary edema, inflammatory cell infiltration, and histopathological damage while lowering serum and bronchoalveolar levels of IL-1β and IL-18. In vitro and in vivo findings demonstrated that emodin suppresses NLRP3 inflammasome-dependent pyroptosis by downregulating NLRP3, ASC, caspase-1, and Gasdermin D cleavage—a mechanism that proves crucial for attenuating inflammatory cell death in lung tissue and macrophages [119]. The same pathway has also been implicated in models of lung ischemia-reperfusion injury and pancreatitis-associated lung injury, further supporting emodin’s broader cytoprotective and anti-inflammatory profile within the respiratory system [120,121].

In rheumatoid arthritis models, emodin attenuates autoimmune inflammation and joint destruction by targeting oxidative stress-driven inflammasome pathways. In collagen-induced arthritis, emodin reduced paw swelling, synovial hyperplasia, inflammatory cell infiltration, and cartilage erosion and lowered systemic levels of pro-inflammatory cytokines. Mechanistic studies identified inhibition of the ROS/TXNIP/NLRP3 inflammasome axis at the spinal cord level as a key event, with emodin disrupting a pathway now considered central to arthritis pathogenesis [122]. Beyond rheumatoid arthritis, emodin also shows chondroprotective and osteoprotective effects in experimental models of joint degeneration and bone loss. In surgically induced degenerative joint disease, intra-articular emodin reduced oxidative stress, suppressed ECM-degrading markers (COMP, CTX-I/II), improved histological cartilage scores, and activated Nrf2/NQO1/HO-1 signaling in cartilage, restoring structural and inflammatory balance within the cartilage microenvironment [123]. In a rat model of post-traumatic knee cartilage degeneration, emodin dose-dependently downregulated MMP-3, MMP-13, and ADAMTS-4 and -5; inhibited interleukin-1β-induced NF-κB and Wnt/β-catenin activation in chondrocytes; and significantly ameliorated structural progression in vivo, supporting its utility as a disease-modifying joint-protective candidate [124]. Complementing these joint-focused effects, emodin inhibits RANKL-induced osteoclastogenesis in bone marrow macrophages and reduces osteoclast-mediated bone resorption by suppressing NF-κB, c-Fos, and NFATc1, thereby regulating bone remodeling in vivo and in vitro [125]. In titanium particle-induced calvarial osteolysis, systemic emodin treatment diminished inflammatory bone loss, decreased osteoclast numbers, and disrupted F-actin ring formation via inhibition of IKKα/β-NF-κB signaling, pointing to potential applications in peri-prosthetic osteolysis and aseptic loosening [126]. Finally, in ovariectomized rats, emodin enhanced BMP-9/Smad-dependent osteogenesis and improved trabecular microarchitecture when combined with low-dose estrogen, suggesting that in osteoporosis it may serve as a bone-anabolic adjuvant to conventional hormone-active therapies [127].

However, within the same therapeutic domain, diacerein stands out as the only clinically validated anthraquinone benchmark for the management of chronic joint inflammation. In vivo, it has been extensively studied as an anti-inflammatory, cartilage- and bone-protective anthraquinone in chronic arthritis models. In a TNF-transgenic Tg197 mouse model of inflammatory polyarthritis, daily oral diacerein (2 mg/kg) delayed disease onset, reduced clinical arthritis scores, and significantly decreased synovitis, cartilage degradation, and bone erosions, with efficacy at the lowest dose surpassing methotrexate in attenuating the TNF-driven progression of arthritis [128]. Parallel osteoarthritis models in dogs and rabbits, and surgically induced knee osteoarthritis in rabbits, showed that diacerein reduces cartilage collagenase activity, preserves non-calcified cartilage thickness, and ameliorates subchondral bone changes, supporting genuine disease-modifying effects on joint structure in vivo [80,129]. These data are mechanistically consistent with diacerein’s metabolism to rhein, which inhibits IL-1β signaling and downstream catabolic mediators in cartilage and synovium while offering more favorable pharmacokinetic and safety profiles than direct administration of rhein [130].

In a multicenter, double-blind, placebo-controlled pilot trial in methotrexate-inadequate responder rheumatoid arthritis patients, add-on diacerein (100 mg/day) produced higher EULAR response rates at week 24 (75% vs. 25%) and, in patients followed to week 28, improved ACR20 responses (81% vs. 47%), with a safety profile comparable to placebo apart from expected chromaturia [131]. A systematic meta-analysis of randomized controlled trials evaluated the efficacy and safety of diacerein in knee and hip osteoarthritis, drawing on data from electronic database searches, manual literature review, and unpublished manufacturer reports. Across nineteen studies, diacerein demonstrated significant superiority over placebo during active treatment, with efficacy and tolerability comparable to nonsteroidal anti-inflammatory drugs (NSAIDs). Notably, unlike NSAIDs, diacerein exhibited a sustained carryover effect, with analgesic-sparing benefits persisting for up to three months after treatment cessation, supporting its role as a symptom-modifying therapeutic option in osteoarthritis management [132].

### 6.4. Therapeutic Potential in Metabolic and Cardiovascular Disorders

Obesity, insulin resistance, and atherosclerosis arise from a chronic low-grade inflammatory milieu in adipose tissue, liver, and vasculature that is tightly coupled to disordered lipid and glucose metabolism. Within this context, emodin and related anthraquinones act as immunometabolic modulators, reshaping inflammatory and energy-sensing pathways in both preclinical and, for some congeners, early clinical studies of obesity and type 2 diabetes.

In high-fat diet (HFD)-induced obesity, emodin improves both adiposity and systemic metabolic parameters in vivo. In obese C57BL/6J mice, repeated oral emodin treatment reduced body weight gain and food intake, improved oral glucose tolerance, and lowered circulating lipids while remodeling white and brown adipose tissue toward a more thermogenic and metabolically active state. Emodin induced the “beiging” of subcutaneous white adipose tissue and increased the expression of beige/BAT markers such as UCP1, CD36, FATP4, PPARα, and prohibitin in subcutaneous WAT and BAT, alongside a selective remodeling of phospholipid and sphingolipid species in adipose depots [100]. Earlier work in HFD-fed rats showed that emodin reduced epididymal fat mass and activated AMPK and its downstream target acetyl-CoA carboxylase (ACC) in white adipose tissue, upregulated carnitine palmitoyl transferase 1 (CPT 1), and suppressed S sterol regulatory element binding protein 1 (REBP 1) and fatty acid synthase (FAS), collectively promoting fatty acid oxidation and limiting lipogenesis [133]. 

A systematic review and meta-analysis of emodin in animal models of type 2 diabetes further supports significant improvements in fasting glucose, glucose tolerance, and insulin sensitivity, together with reductions in total cholesterol, triglycerides, and LDL cholesterol and the partial normalization of HDL cholesterol [99]. These metabolic benefits are repeatedly linked to the upstream control of AMPK-centered energy-sensing pathways and the mitigation of inflammation-driven metabolic derangement, reinforcing the view of emodin as a potential pleiotropic metabolic modulator rather than a stand-alone hypoglycemic drug [99,101]. In addition, emodin co-treatment dose-dependently attenuated rosiglitazone-induced weight gain, visceral adiposity (iSAT and eWAT mass; *p* < 0.05), and ectopic lipid accumulation in mice while simultaneously improving hepatorenal dysfunction. Notably, the anthraquinone synergistically enhanced the glucose-lowering efficacy of the reference drug. These effects were associated with the suppression of SREBP1-driven lipogenesis (Srebp1, Acc, Fasn, Scd1; *p* < 0.05) and the potentiation of thermogenic programming through enhanced PPARγ nuclear translocation and transcriptional activity [134].

Among cardiovascular indications, atherosclerosis has emerged as a particularly compelling target for emodin. Emodin significantly attenuated atherosclerotic lesion formation in high-fat-diet-fed ApoE^−^/^−^ mice, concomitant with a marked suppression of inflammasome- and pyroptosis-related markers, including NLRP3, GSDMD, IL-1β, and IL-18. Consistent effects were observed in PMA-stimulated macrophages, where emodin inhibited NF-κB activation by disrupting TLR4/MyD88 complex formation [135].

Within the same chemical family, several anthraquinone congeners show metabolic efficacy that in specific settings matches or exceeds that of emodin. Rhein is particularly well documented in diabetic nephropathy and broader glycolipid dysregulation. In streptozotocin-induced and db/db diabetic models, rhein reduces fasting glucose and lipids, attenuates albuminuria and glomerulosclerosis, and suppresses renal inflammation and fibrosis by acting on TGF β/Smad, NF κB, and AMPK-related pathways [136]. A meta-analysis of rhein in diabetic kidney disease reports significant improvements in renal function markers and metabolic indices, strengthening its profile as a renoprotective, metabolically active agent [137]. More recent work indicates that rhein inhibits ferroptosis and epithelial-mesenchymal transition in diabetic nephropathy via modulation of Rac1/NOX1/β-catenin signaling, improving histological outcomes in diabetic mice [138]. 

Diacerein, the acetylated prodrug of rhein, has progressed furthest clinically for metabolic control in type 2 diabetes. In a randomized, placebo-controlled trial in drug-naïve patients with T2DM, daily diacerein intake for three weeks reduced HbA1c and fasting glucose and significantly improved first- and second-phase insulin secretion while lowering circulating IL 1β and TNF α, consistent with the anti-inflammatory restoration of β cell function [107]. Subsequent trials and a meta-analysis indicate that diacerein, either alone or added to metformin, produces modest but clinically relevant improvements in glycemic control and some cardiometabolic parameters, though heterogeneity in lipid and insulin resistance outcomes remains [139,140].

Other hydroxyanthraquinones have demonstrated similar metabolic benefits. In an experimental study on free anthraquinones from rhubarb (including aloe-emodin, rhein, emodin, chrysophanol, and physcion) in HFD-fed rats, emodin and rhein exhibited the strongest effects in attenuating weight gain, visceral fat accumulation, adipocyte hypertrophy, and inflammatory cytokine expression. At 50 μM, each reduced intracellular triglyceride levels by approximately 30% in differentiated cells and increased glycerol release, indicating enhanced lipolysis. Despite their structural similarity, the two compounds engaged distinct metabolic mechanisms: rhein suppressed adipogenesis by downregulating adipogenic transcription factors and activating lipolytic pathways, whereas emodin primarily reduced lipogenic enzyme expression via MAPK-dependent signaling [141]. A recent review on anthraquinones in insulin resistance highlights that aloe-emodin and emodin activate AMPK in liver and skeletal muscle, enhancing fatty acid oxidation and suppressing lipogenesis, while chrysophanol and physcion promote energy expenditure and thermogenic gene expression in brown and beige adipose tissue in high-fat-diet models [101]. In preclinical studies, several 1,8-dihydroxyanthraquinones exhibit antihyperglycemic activity, with aloe-emodin showing pronounced glucose-lowering effects in experimental models; however, direct intestinal α-glucosidase inhibition appears to be more consistently associated with emodin, particularly in biochemical assays and animal studies relevant to postprandial glucose regulation [7].

### 6.5. Therapeutic Potential in Neurodegenerative Diseases

The neuroprotective activity of anthraquinones has been demonstrated across several preclinical models, but the evidence is still emerging and focused on acute injury and neuroinflammation, with fewer data in chronic neurodegenerative diseases such as Alzheimer’s and Parkinson’s disease. Of note, most of these studies are limited by short treatment durations, relatively high dosing regimens, and an incomplete assessment of long-term functional, behavioral, and disease-modifying outcomes. 

Among the reviewed anthraquinones, emodin’s neuroprotective profile is currently strongest, demonstrating consistent efficacy in reducing tissue injury across experimental models of acute neuroinflammation. Its role in chronic neurodegenerative diseases is intriguing but still preliminary, with Parkinson’s disease models providing the first in vivo proof-of-concept that warrants longer-term investigations.

The intraperitoneal administration of emodin in a rat model of experimental autoimmune encephalomyelitis (EAE) significantly ameliorated disease severity, reducing body weight loss, neurobehavioral deficits, inflammatory infiltration, and demyelination. These effects were mechanistically linked to the suppression of microglial aggregation and activation and the downregulation of pro-inflammatory cytokine production and NLRP3 inflammasome signaling in the central nervous system [142]. Consistent with its antioxidant and anti-apoptotic properties, emodin also exerted neuroprotection in the settings of acute ischemic and hypoxic brain injury. In a rat middle cerebral artery occlusion model, it reduced infarct volume, improved neurological function, decreased ROS generation and glutamate release, and shifted the Bcl-2/active caspase-3 balance toward ERK1/2-driven cell survival [143]. Similarly, in a Vannucci model of hypoxic-ischemic injury in neonatal mice, emodin lowered infarct size, brain edema, neuronal apoptosis, and degeneration, and it facilitated the recovery of brain tissue morphology by inhibiting the expression of the proapoptotic factors p53, Bax, and caspase-3 [144].

More recently, toxin-based Parkinson’s disease models have suggested that emodin might also confer protection in dopaminergic neurodegeneration, with ferroptosis and redox signaling as key mechanistic themes. In an MPTP-induced Parkinson mouse model, emodin improved motor and cognitive performance, reduced dopaminergic neuronal loss in the substantia nigra, and decreased malondialdehyde and other oxidative stress markers while upregulating Nrf2 and its downstream antioxidant targets [145]. A complementary study by Yusun et. al. found that emodin protects dopaminergic neurons by suppressing ferroptosis, as demonstrated in Erastin- and MPP^+^-challenged models of Parkinsonian neurotoxicity. The authors reported attenuation of lipid peroxidation and iron-dependent cell death signaling through upregulation of the mitochondrial complex III component UQCRC1, identifying a novel target in emodin’s neuroprotective effects [146].

Besides emodin, several related anthraquinones have likewise shown promising neuroprotective potential in preclinical studies. In experimental models of Alzheimer’s disease, the closely related aloe-emodin produced encouraging results by inhibiting β-amyloid aggregation and attenuating Tau hyperphosphorylation—two central pathological hallmarks of disease progression. Similarly, in Parkinson’s disease models, it preserved dopaminergic neuron viability against neurotoxin-induced apoptosis, broadly mirroring emodin’s anti-oxidative and anti-inflammatory profile [68]. 

Rhein also displays convergent neuroprotective actions in animal models across several acute and chronic CNS pathologies, largely by attenuating inflammatory and oxidative cell-death pathways. In a traumatic brain injury model, it lessens neurological deficits and tissue damage by suppressing caspase-1-dependent pyroptosis and associated cytokine release [147]. In Alzheimer’s disease-relevant models, rhein improves behavior and mitigates microglia-driven neuroinflammation by rewiring glutamine-aspartate-arginine metabolism, downregulating GLS1/GOT1, and thereby limiting nitric oxide production [148]. In an experimental model of ischemic stroke, rhein dose-dependently improved neurological scores, reduced infarct volume and preserved blood–brain barrier integrity through activation of the NRF2/SLC7A11/GPX4 axis and limiting oxidative stress and ferroptosis [149]. Finally, in preclinical PTZ-induced epilepsy, rhein delayed seizure onset; reduced seizure severity, duration, and frequency; and ameliorated associated neurological deficits, concomitant with the inhibition of TLR4-NF-κB signaling [150].

## 7. Safety, Toxicity, and Limitations

Despite extensive preclinical evidence supporting the biological activity of emodin-type anthraquinones, their safety profile and pharmacological liabilities may still pose substantial barriers to therapeutic development. In experimental settings, emodin has demonstrated a clear dose-dependent toxicity profile with prominent gastrointestinal, hepatic, renal, and potential genotoxic liabilities. These risks reflect a shared structural liability of emodin-type anthraquinones rather than an idiosyncratic property of emodin alone. Indeed, it has become clear that the same chemical features that underpin the broad spectrum of bioactivities of these compounds (a redox-active quinone system and multiple phenolic hydroxyls) also confer liabilities that can emerge under high exposure, prolonged use, or in vulnerable populations. However, most toxicological signals arise from in vitro systems that are not readily extrapolatable to the macroorganism level and/or involve supratherapeutic concentrations far exceeding those achievable in humans. The translation of current safety data to the clinical context is therefore constrained by limited in vivo pharmacokinetic data and the absence of well-designed human trials. In addition, accumulating in vivo evidence indicates that organ toxicity is a context-dependent phenomenon, in which emodin-type anthraquinones may exert harmful effects under excessive exposure but activate adaptive, stress-responsive protective pathways under conditions of acute tissue injury, inflammation, or metabolic stress.

### 7.1. Dose-Dependent Toxicity

Across preclinical models, emodin toxicity is strongly dose- and time-dependent, with a relatively wide margin between low micromolar concentrations used for mechanistic signaling studies and higher exposures that trigger cell death or organ injury. In rodent in vivo studies, Sougiannis et. al. demonstrated that subchronic (12-week) exposure to emodin up to 80 mg/kg did not elicit overt injury in major organs [151], whereas repeated dosing at ≥80–150 mg/kg or gram-per-kg anthraquinone-rich extracts induced histopathological changes in the liver, kidney, and reproductive organs. However, the LD_50_ of oral anthraquinone (unsubstituted anthracene-9,10-dione) administered to SD rats was estimated to be >5000 mg/kg [152]. The National Toxicology Program (NTP) 2-year feed study in F344/N rats and B6C3F1 mice exposed animals to 280–2500 ppm emodin, revealing dose-dependent renal-tubule hyaline droplet accumulation and pigmentation but no clear carcinogenic activity in male rats, with only equivocal evidence (marginal Zymbal’s gland carcinoma increase) in female rats. Lethality and marked clinical toxicity occurred only at the highest short-term doses (e.g., 17,000–50,000 ppm in 16-day studies), where strong weight loss, decreased feed intake, and macroscopic kidney and gallbladder lesions were evident [153].

In vitro, many cancer cell studies report cytotoxic and pro-apoptotic effects at 20–100 µM, but normal cell toxicity (e.g., hepatocytes, renal tubular cells, cardiomyocytes) frequently emerges in a similar or only slightly higher range, indicating a narrow selectivity window [84]. Such concentrations generally exceed free plasma levels achievable after oral dosing in rodents due to low bioavailability (~3.2% absolute oral bioavailability) and extensive glucuronidation, suggesting that much of the in vitro toxicity literature exaggerates on-target risk relative to realistic systemic exposures. Conversely, pharmacokinetic studies in rats indicate that limited intestinal absorption can result in substantially higher luminal concentrations of emodin following oral administration (approximately 56% of the administered dose recovered unchanged in feces), which likely contributes to the pronounced local gastrointestinal effects associated with chronic anthraquinone exposure [39].

### 7.2. Gastrointestinal Toxicity

Anthraquinones are historically associated with stimulant laxative activity, and emodin is no exception. In vivo work directly connects emodin to increased fecal water content and defecation indices, mechanistically linked to aquaporin-3 (AQP3) regulation in the colon and signaling via PKA/p-CREB in a mouse model and in HT-29 cells [154,155]. While these findings are sometimes discussed as therapeutically useful for constipation, they also pose a safety concern: at higher doses, gastrointestinal cramping, diarrhea, dehydration, and electrolyte imbalance become plausible, especially with chronic or abusive use of anthraquinone-containing products [43,156]. Long-term anthraquinone laxative use is linked to melanosis coli, a pigmentation change associated with chronic exposure to stimulant laxatives; however, beyond pigmentation, the larger concern is whether chronic anthraquinone exposure is associated with colorectal neoplasia [156].

Dedicated in vivo studies, as well as epidemiological data, suggest a 2–3-fold increase in colorectal cancer risk with long-term anthraquinone laxative use (senna, cascara, rhubarb), although much of the evidence derives from complex plant preparations rather than a pure compound [157,158]. Conversely, a recent systematic review and meta-analysis of randomized controlled trials and observational studies reported an increased odds ratio that did not reach conventional statistical significance (pooled OR 1.4–1.5), but the directionality sustains ongoing concern and the need for the careful interpretation and evaluation of confounding factors, as chronic constipation itself may increase colorectal cancer risk [159].

### 7.3. Hepatotoxicity vs. Hepatoprotection

Anthraquinone derivatives exhibit two-way hepatic responses, combining context-dependent hepatotoxicity and robust hepatoprotective effects in various drug-induced liver injury models. Emodin has been repeatedly shown to exert concentration- and time-dependent cytotoxicity in normal hepatocytes and hepatoma-derived systems, with mitochondrial dysfunction emerging as a proximal trigger for apoptotic cell death. Emodin can also accumulate within mitochondria and promote cyclophilin D-associated mitochondrial permeability transition, further amplifying mitochondrial dysfunction and apoptosis [3]. Importantly, the CYP1A-mediated biotransformation of emodin to 5-hydroxyemodin generates a more hepatotoxic metabolite that activates the aryl hydrocarbon receptor (AhR), enhances ROS production, triggers ER stress (ATF4/CHOP), and amplifies mitochondrial damage, while AhR inhibition or CYP1A blockade mitigates injury [5]. Interference with major clearance routes (extensive glucuronidation via UGT1A1/UGT1A9/UGT2B7 and MRP2-mediated efflux) also increases emodin exposure and liver toxicity, as established for probenecid co-treatment [160,161]. Hepatotoxicity is also aggravated upon CYP3A induction and glutathione depletion, leading to extensive oxidative and mitochondrial stress [162].

Paradoxically, the same anthraquinone moiety has been reported to exert hepatoprotective effects under toxicant challenge in multiple injury models. Emodin pretreatment (15–30 mg/kg) attenuated acetaminophen-induced hepatotoxicity in mice and reduced ALT/AST levels by restoring GSH, limiting lipid peroxidation, upregulating Nrf2/HO-1/NQO1, inhibiting NLRP3 inflammasome activation and suppression of cGAS-STING-IFN signaling [163]. Rhein, while capable of CYP2C19-dependent bioactivation to a hepatotoxic reactive epoxide metabolite [164], also protects against methotrexate-induced liver injury by Nrf2-HO-1 activation, restoration of Bcl-2/bax balance, and inhibition of NF-κB/TNF-α/Caspase-3 signaling [165]. Diacerein, the prodrug of rhein, similarly counteracts acetaminophen- and amiodarone-induced hepatotoxicity in rodents via antioxidant activity, suppression of HMGB1/TLR4/NF-κB and TLR4/NF-κB/NLRP3 pathways, attenuation of apoptotic signaling, and enhancement of PPAR-γ activity [166,167,168].

### 7.4. Nephrotoxicity vs. Nephroprotection

The renal effects of anthraquinone derivatives are similarly biased, as both nephrotoxicity and nephroprotection are reported depending on dose, exposure duration, metabolic context, and injury model. Emodin, the most extensively studied member, has been shown to induce nephrotoxicity under conditions of high dose or prolonged exposure, primarily through oxidative stress-driven tubular injury and apoptotic cell death. In vivo and in vitro studies demonstrate that emodin elevates serum creatinine and blood urea nitrogen, disrupts mitochondrial membrane potential, and depletes glutathione in renal tubular epithelial cells (NRK-52E), culminating in ferroptosis and apoptosis. Inhibition of the Notch1/Nrf2/GPX4 antioxidant axis leads to iron accumulation, ROS overproduction, and lipid peroxidation, while pharmacological activation markedly attenuates these toxic effects [169]. Additional evidence suggests that chronic exposure to rhubarb extracts rich in anthraquinones preferentially targets renal tubular epithelial cells, with inflammation-associated cytokines (TNF-α, TGF-β1, IL-6, MCP-1) and disruption of the glomerular filtration barrier contributing to nephrotoxic outcomes [1].

Paradoxically, emodin also exhibits robust nephroprotective activity in multiple injury models. In cisplatin-induced acute kidney injury (AKI), 10 mg/kg oral administration of emodin significantly ameliorated tubular necrosis, oxidative stress, and apoptotic signaling without altering renal cisplatin accumulation, largely by restoring antioxidant enzyme activity and suppressing ROS generation [170].

Aloe-emodin similarly exhibits context-dependent renal effects. On one hand, it can induce hepatotoxicity and renal stress by inhibiting MRP2 and disturbing redox homeostasis in hepatocytes [171], yet in cisplatin-induced AKI models it acts as a ferroptosis inhibitor that improves renal function, reduces oxidative stress markers, limits ferroptosis, and activates Nrf2 and its downstream antioxidant genes. Notably, Nrf2 blockade abrogates this protection, confirming a Nrf2-dependent renoprotective mechanism [172].

Rhein has demonstrated predominantly renoprotective activity in vivo, with extensive data indicating benefit in diverse kidney injury models, whereas reported nephrotoxicity is mostly confined to high-dose in vitro systems and appears to be strongly dependent on dose and duration [173]. Reactive metabolite formation via the CYP2C19 isoenzyme appears to be engaged in nephrotoxicity in addition to hepatotoxicity, as CYP2C19 inhibition normalizes liver transaminase levels [164,174]. In contrast, rhein alleviates cisplatin-induced AKI by downregulating NOX4-driven oxidative stress and NF-κB-mediated inflammation, without impairing the antitumor efficacy of the cytostatic drug [175]. Finally, diacerein, a clinically used semi-synthetic anti-inflammatory anthraquinone, consistently demonstrates nephroprotective effects. In models of cisplatin- and diclofenac-induced nephrotoxicity, diacerein restores renal function, suppresses oxidative stress, and inhibits apoptosis via activation of Nrf2/HO-1 and SIRT1-dependent regulatory axes, alongside repression of NF-κB, HIF-1α, and p53 signaling [176,177].

### 7.5. Genotoxicity and Reproductive Effects

Overall observations indicate that anthraquinone geno- and reproductive toxicity is highly conditional, emerging under particular structural, dosing, and assay conditions, while many recent in vivo studies fail to corroborate a consistent hazardous risk.

Early work on hydroxyanthraquinones highlighted a clear genotoxic potential under certain conditions, particularly for emodin, aloe-emodin, danthron, and other structurally related congeners. In mammalian systems, emodin, aloe-emodin, and danthron inhibited topoisomerase II in cell-free decatenation assays and interfered with bisbenzimide-DNA binding, producing chromosomal lesions in mouse lymphoma L5178Y cells that closely resembled those induced by classic topoisomerase II poisons such as etoposide and m-amsacrine [178]. In broader test batteries, only hydroxyanthraquinones with specific structural features—1,3-dihydroxy substitution (e.g., emodin, purpurin) or a hydroxymethyl side chain (e.g., lucidin, aloe-emodin)—were mutagenic in V79 HGPRT, DNA-repair, and C3H/M2 transformation assays, establishing a strong structure–activity relationship in their genotoxicity profile [179]. Consistently, emodin, aloe-emodin, and danthron induced tk mutations and micronuclei in L5178Y cells, with danthron > aloe-emodin > emodin in potency, and all three inhibited topoisomerase II-mediated decatenation in vitro, supporting topoisomerase II inhibition as a central mechanism [180]. However, in more physiologic food matrices, emodin was clearly genotoxic in comet, micronucleus, and mutation assays, whereas chrysophanol and physcion were negative; whole-vegetable extracts containing these anthraquinones failed to induce micronucleus formation and in some cases even attenuated danthron-induced micronuclei, indicating that co-occurring phytochemical constituents can substantially modulate genotoxic outcomes [23].

More recent evaluations have yielded heterogeneous and often negative findings in vivo, complicating risk interpretation. A substance-based medical device containing anthraquinones did not increase micronucleus frequency in TK6 cells in an OECD 487-conform flow-cytometric assay and instead reduced intracellular ROS, indicating an absence of clastogenic/aneugenic activity under the tested conditions [181]. Purified anthraquinone itself (unsubstituted anthracene-9,10-dione) was non-mutagenic in standard Ames, mammalian erythrocyte micronucleus, and in vitro chromosome aberration assays, although high-dose prenatal exposure in rats produced maternal toxicity and increased visceral malformations, with a NOAEL of 21.76 mg/kg and LOAEL of 217.6 mg/kg [182]. In vivo comet assays have also challenged earlier concerns: aloe-emodin up to 2000 mg/kg in mice showed no increase in DNA strand breaks in the kidney or colon, aside from a possible oxidative component after repair-enzyme treatment [183], and a purified Aloe vera whole-leaf dry juice was negative in both mouse lymphoma TK and rat comet assays [184]. Likewise, rhubarb rhizome extract (total hydroxyanthracenes 1.39%, predominantly rhein, physcion, chrysophanol) showed no mutagenic or clastogenic activity in Ames or human lymphocyte micronucleus assays [185].

Injurious effects of anthraquinones on mammalian reproduction and embryonic development have also produced controversial results. In vitro exposure of mouse oocytes to emodin (40 µM) significantly decreased fertilization rates and cleavage to two-cell and blastocyst stages, and it induced apoptosis in both inner cell mass (ICM) and trophectoderm (TE) cell populations. Embryo transfer studies showed that blastocysts derived from emodin-pretreated oocytes displayed lower implantation rates, higher resorption rates, and reduced fetal weights compared to controls—effects reversible by caspase-3 inhibitor co-treatment, confirming an apoptosis-mediated teratogenic mechanism [186]. However, an NTP developmental toxicity study found that prenatal mortality, live litter size, fetal sex ratio, and morphological development were unaffected in rats and mice at moderate doses, with only high-dose maternal weight reduction observed in rats [187].

### 7.6. Interactions with Drug-Metabolizing Enzymes and Transporters

Interactions between anthraquinones and biotransformation systems are mechanistically important but not unique to this phytochemical class: many plant-derived constituents and even common dietary factors can modulate cytochromes P450, conjugating enzymes, and membrane transporters, with implications for herb–drug interactions and resistance phenotypes. 

Emodin and its congeners nonetheless show a particularly rich interaction profile. Emodin inhibits several CYP isoforms in the low-micromolar range in vitro (IC_50_ CYP1A2 3.7 µM, CYP1A1 12.3 µM, CYP2B1 14.9 µM) and downregulates key UGTs while also acting on efflux pumps such as MRP2, raising the potential for the altered clearance of co-medications [188]. Related anthraquinones exhibit distinct CYP regulation: physcion competitively suppresses CYP2C9 and CYP2D6 (IC_50_ 7.44 and 17.84 µM; respectively) and non-competitively inhibits CYP3A4 in a time-dependent manner (IC_50_ 13.5 µM), suggesting clinically relevant interaction potential at sufficient exposure [189]. However, circulating free concentrations after oral dosing are usually far below these IC_50_ values, whereas intestinal and enterocyte exposures are likely much higher; thus, clinically meaningful drug interactions are more plausible at the level of the gut wall and biliary/renal transport than via systemic CYP inhibition and will depend critically on formulation and dose. Aloe-emodin and chrysophanol are submicromolar non-competitive inhibitors of CYP1B1 (IC_50_ 0.28 and 0.34 µM), whereas rhein is much weaker (IC_50_ ≈ 23.7 µM), with inhibition strength closely tied to hydroxyl and methoxy substitution patterns. These structure–activity insights have been explored to design CYP1B1-directed antitumor approaches, particularly in hormone-responsive malignancies [190].

Anthraquinones also interact with drug transporters, particularly P-glycoprotein (P-gp) and multidrug resistance-associated proteins (MRPs). Chamber studies identified emodin as both a substrate and modulator of P-gp and MRP2 and MRP3 in rat enterocytes, implying complex effects on intestinal absorption and biliary/renal excretion [4]. Importantly, transporter modulation has been leveraged therapeutically in oncology: emodin, aloe-emodin, rhein, diacerein, and related derivatives consistently reverse multidrug resistance by inhibiting P-gp efflux and/or downregulating its expression, thereby enhancing intracellular drug accumulation and chemosensitivity across leukemia, lung, breast, pancreatic, and ovarian cancer models [191,192,193,194,195].

## 8. Strategies to Improve Therapeutic Utility

Formulational strategies to improve the therapeutic utility of anthraquinones focus on overcoming intrinsic liabilities such as poor solubility, rapid first-pass metabolism, low oral bioavailability, and off-target toxicity. Approaches span from nanoemulsions, nanocrystals, and micelles to cocrystals, solid dispersions, prodrug/ester design, and targeted or hybrid delivery systems and have been applied most extensively to emodin, aloe-emodin, rhein, and diacerein. Across these platforms, the common strategy is to decouple pharmacodynamic potential from ADME constraints, allowing anthraquinones to be employed at lower, safer systemic exposures while achieving higher local or target-site concentrations. Parenteral delivery of anthraquinone formulations (intravenous, subcutaneous, or intramuscular) bypasses first-pass metabolism and can achieve significantly higher systemic availability, but its broader use is constrained by the intrinsic poor aqueous solubility of these compounds and the associated risk of local injection-site irritation or injury [196].

For emodin, a key barrier is extensive UGT-mediated glucuronidation. A Cremophor EL-based nanoemulsion (EMO-NE, ~116 nm) increased apparent permeability 2.3-fold in UGT1A1-overexpressing MDCKII monolayers and reduced total glucuronide formation by ~57%. As reported, Cremophor EL acted as a mixed-type UGT1A1 inhibitor and enhanced transcellular transport; however, its well-documented hypersensitivity reactions and tolerability concerns in paclitaxel formulations may limit clinical applicability despite its favorable pharmacokinetic effects [6].

A complementary strategy uses an emodin-nicotinamide cocrystal (ENC) combined with polyvinylpyrrolidone K30 (PVP) as a crystallization inhibitor and solubilizing agent. In simulated intestinal fluid containing 1.5% PVP, the ENC cocrystal achieved roughly twice the solubility of emodin, but this advantage did not translate into higher parent-drug exposure in rats. Rather, systemic levels of the main emodin metabolite mimicked the in vitro dissolution behavior, indicating that emodin undergoes rapid gastrointestinal metabolism during the absorption phase and concurrent metabolic inhibition may be needed to vitally improve parent drug bioavailability in this particular model [35]. Beyond single formulations, a recent review synthesizes progress on emodin-loaded nano-drug delivery systems (liposomes, polymeric micelles, nanoparticles, nanogels), showing that nanocarriers often improve solubility, pharmacokinetics, tumor accumulation, and antitumor efficacy across breast, liver, and lung cancer models while mitigating systemic toxicity [2]. Disease-tailored delivery systems, including mannose-decorated, colon-targeted micelles incorporating emodin, have demonstrated enhanced oral bioavailability, improved local retention, and accelerated mucosal healing in experimental ulcerative colitis models. More recently, carbon-based emodin-derived nanoplatforms (carbon dots) have been developed with red-emissive and enzyme-mimetic properties, showing potent reactive oxygen species-modulating activity and therapeutic efficacy in preclinical models of oxidative injury and sepsis-related organ damage. While these carbon nanomaterials exhibit improved solubility and biocompatibility compared to conventional heavy-metal quantum dots, their long-term safety, biodistribution, and clinical translatability remain to be systematically evaluated [197]. Pulmonary emodin nanocrystals have been explored for mucus-penetrating delivery in bleomycin-induced fibrosis [198], and deoxycholic-acid-chitosan-coated liposomes combined with in situ colonic gels have been developed to improve renal uptake and restore microbiota dysbiosis in renal fibrosis [199].

Aloe-emodin has also been upgraded through diverse formulation and chemical-modification routes. Soluplus/glycyrrhizic-acid mixed micelles (~30 nm) increase oral bioavailability by 3.1-fold, accelerate dissolution in multiple media, and enhance anti-hyperuricemic and anti-inflammatory effects in gouty arthritis rats by more efficiently inhibiting xanthine oxidase and lowering IL-1/IL-6 levels [200]. Solid lipid nanoparticles (~89 nm, EE ≈ 98%) provide sustained release and enhance cellular uptake and apoptosis in MCF-7 and HepG2 cancer cells while exhibiting comparatively lower cytotoxicity toward normal mammary epithelial cells in vitro, indicating improved cancer cell selectivity [201]. Solid dispersions of aloe-emodin with PVP and poloxamer convert the drug into an amorphous state, boost dissolution, accelerate absorption, and improve bioavailability without adding acute toxicity [202]. In parallel, rational structural modification has led to the development of N-heterocycle-containing aloe-emodin derivatives, exemplified by compound 12r. This analogue features 1,8-dimethoxy substitution of the anthraquinone core and a C-3 methylene linker conjugated to a piperazine moiety bearing a terminal bis(4-fluorophenyl)methyl group, designed to enhance lipophilicity and optimize molecular interactions. While preserving the essential anthraquinone pharmacophore, this modification markedly improves pharmacokinetic performance, achieving approximately 55% oral bioavailability. Biologically, compound 12r demonstrates pronounced suppression of nitric oxide and pro-inflammatory cytokine production, along with significant therapeutic efficacy in experimental models of ulcerative colitis [203]. Peptide-conjugated aloe-emodin further improves water solubility and selectively targets HER2-positive SKBR3 cells with predominant nuclear accumulation and greater cytotoxicity than the parent compound [204].

Technological approaches for diacerein seek to increase early absorption (reducing unabsorbed colonic drug available for conversion to rhein) and/or sustain delivery and improve systemic exposure. Diacerein cocrystals with nicotinamide, isonicotinamide, or theophylline increase intrinsic dissolution and enhance anti-arthritic activity relative to the parent drug, illustrating how crystal engineering can address solubility-limited bioavailability [205]. More complex systems include chitosan-coated diacerein nanosuspensions, which raise oral bioavailability by ~170% and lower intraluminal rhein formation and diarrhea in rats [206]; high-load self-nanoemulsifying nanosuspensions that more than double rhein bioavailability and exploit lymphatic uptake [207]; and ternary solid dispersions embedded in asymmetric osmotic-pump tablets that yield zero-order 24 h release and nearly three-fold-higher systemic exposure [208]. Orally disintegrating tablets built on a solid-dispersion DCN system further accelerate dissolution and onset, improving early anti-inflammatory effects while aiming to limit colonic rhein formation [209]. For rhein itself, β-cyclodextrin conjugation markedly increases aqueous solubility and cytotoxic potencial against HeLa cells [210]. Solid lipid nanoparticles double its oral bioavailability [211], and rhein-decorated fibrin gels or methotrexate-co-loaded SLNs enable local, sustained release to intervertebral discs or inflamed joints, enhancing efficacy and minimizing systemic exposure [212,213].

Notably, glycosylation-based scaffold engineering has emerged as an effective strategy to amplify intrinsic cytotoxic potency. A series of anthracene L-rhamnopyranoside derivatives of emodin were designed to exploit the sugar-mediated modulation of solubility, membrane interaction, and intracellular trafficking. Among these, the di-rhamnosylated derivative EM-d-Rha (S-8; 3-(2″,3″-di-O-acetyl-α-L-rhamnopyranosyl-(1→4)-2′,3′-di-O-acetyl-α-L-rhamnopyranosyl)-emodin) displayed markedly enhanced antiproliferative activity across a broad panel of human cancer cell lines, including lung (A549), liver (HepG2), ovarian (OVCAR-3), cervical (HeLa), leukemia (K562), and gastric (SGC-790) models. IC_50_ values were consistently in the low-micromolar range and approximately ten-fold lower than those of parent emodin, demonstrating that scaffold glycosylation can yield substantial shifts in both antitumor spectrum and potency [214].

## 9. Conclusions

The totality of evidence reviewed here positions emodin as the most extensively characterized member of the anthraquinone scaffold, with a breadth of reproducible preclinical efficacy that spans malignant, inflammatory, and immunometabolic disease domains. At the same time, translational progress has been constrained by a convergence of ADME and safety issues. Emodin’s poor aqueous solubility, extensive first-pass glucuronidation, and efflux lead to low and variable oral bioavailability, while high luminal exposures in the colon create a mismatch between local and systemic safety profiles. From a development perspective, the modulation of drug-metabolizing enzymes and transporters represents a double-edged sword, simultaneously raising concerns regarding herb–drug interactions while offering mechanistic opportunities for overcoming multidrug resistance in oncology.

Against this backdrop, the most credible path forward lies in re-engineering the biopharmaceutical properties of emodin rather than further expanding indication space using conventional dosing paradigms. Preclinical studies suggest that innovative formulation platforms, such as nanoemulsions, micelles, solid dispersions, and targeted delivery systems, have the capacity to improve solubility and permeability, attenuate first-pass metabolism, and preferentially enhance drug accumulation in diseased tissues. For the broader anthraquinone scaffold, including rhein/diacerein and aloe-emodin, similar principles apply; that is, translational success will likely come from exposure-aware designs, enabling indication-specific optimization rather than one-size-fits-all extrapolation from in vitro potency.

Future research should prioritize human-relevant pharmacokinetic–pharmacodynamic mapping that explicitly links formulation-controlled exposure profiles to both efficacy and toxicodynamic endpoints. Equally important is the systematic delineation of dose thresholds that separate organ-protective from organ-injuring effects across the liver, kidney, and gastrointestinal systems. By doing so, emodin and its congeners can move from broadly active but “difficult” natural products to carefully engineered agents, whose pleiotropic beneficial properties offer a potential advantage over existing therapies.

## Figures and Tables

**Table 1:** Summary of reported pharmacokinetic parameters of emodin in preclinical models.

Study/Design	Species/Model	Dose/Route	Key PK Parameters	Main Metabolites/Notes	Ref.
Shia et al., J Pharm Sci (2010) [45] Comparative IV vs. PO disposition (parent vs. hydrolyzed samples); focuses on exposure to conjugates.	Sprague-Dawley rats	IV 5 mg/kg; PO 20 and 40 mg/kg	Reported extremely low oral systemic exposure and extensive first-pass metabolism; plasma emodin quantifiable only for a few hours after dosing.	After IV: rapid decline of parent compound and its metabolites (glucuronides + ω-hydroxyemodin). After PO: glucuronides predominated in serum; parent compound often not detected.	[45]
Liu et al., J Pharm (2011) [46] Gender-dependent PK of emodin and emodin-3-O-glucuronide in animal models.	Sprague-Dawley rats, male vs. female	4 mg/kg i.v. emodin	Two-compartment model. Male: t_1/2α_ 13.26 ± 6.28 min, t_1/2β_ 187.38 ± 174.52 min, AUC_0→∞_ 422.71 ± 163.40 min·µg/mL, Cl 2.64 ± 0.86 mL/min/kg. Female: t_1/2α_ 13.52 ± 7.28 min, t_1/_2β 118.50 ± 83.09 min, AUC_0→∞_ 282.52 ± 98.42 min·µg/mL, Cl 3.98 ± 1.56 mL/min/kg.	Reports gender-dependent absolute oral bioavailability. Emphasizes glucuronidation as a major determinant of low systemic exposure. Rapid conversion to emodin-3-O-glucuronide; glucuronide t_1/2Ke_167.40 ± 50.91 min (male) and 251.31 ± 114.20 min (female), AUC_0→∞_ 2210.02 ± 950.09 vs. 1054.42 ± 290.31 min·µg/mL. Supports dominance of conjugated species in vivo.	[46]
Liu et al., 2011, J Pharm (oral arm). [46]	Sprague-Dawley rats, male vs. female	8 mg/kg p.o. emodin	Non-compartmental. Male: C_max_ 0.31 ± 0.094 µg/mL, T_max_ 18.00 ± 6.71 min, AUC_0→∞_ 65.70 ± 34.77 min·µg/mL; absolute F ≈ 1.6–7.5%. Female: C_max_ 0.039 ± 0.011 µg/mL, T_max_ 18.75 ± 7.51 min, AUC_0→∞_ 33.82 ± 4.09 min·µg/mL; absolute F ≈ 0.4–5%.	Emodin-3-O-glucuronide is dominant in plasma: male C_max_ 6.69 ± 1.06 µg/mL, T_max_ 240 min, AUC_0→∞_ 2261.89 ± 655.87 min·µg/mL; female C_max_ 1.81 ± 0.58 µg/mL, T_max_ 60 min, AUC_0→∞_ 458.50 ± 373.29. Total emodin (parent + glucuronide) oral AUC markedly higher in males (3034.59 ± 968.99 vs. 762.07 ± 321.89 min µg/mL).	[46]
Liu et al., Toxicol Appl Pharmacol (2012) [47]	Rat/intestinal models	Mechanistic (UGT/MRP coupling)	Mechanistic PK study focused on intestinal metabolism and transport rather than conventional Cmax/AUC profiling.	Concludes that intestinal UGT metabolism + MRP efflux strongly contribute to poor oral exposure.	[47]
Wang et al., Frontiers; Front Pharmacol. (2021) [37] Review	Multiple	Multiple	Review-level synthesis; useful for contextualizing DDIs (UGT inhibition) and disease-state effects on exposure.	Summarizes that UGT inhibition (e.g., piperine) can raise emodin AUC/Cmax and drug–drug interaction risk); disease states can alter the PH profile of anthraquinones.	[37]
Li et al., Biomed Pharmacother (2017) [48] Rhubarb anthraquinone extract (PK study in physiological and pathological experimental conditions).	Sprague-Dawley rats; normal, diabetic nephropathy, and CCl_4_ liver-injury models	Oral rhubarb anthraquinone extract 37.5, 75, 150 mg/kg (emodin one of four PK markers)	Emodin plasma AUC and C_max_ increased in acute liver injury rats vs. controls; no major PK change in diabetic nephropathy rats (exact numeric values for emodin not fully detailed in abstract).	Emodin measured alongside rhein, aloe-emodin, chrysophanol, and physcion; data support disease-dependent changes in exposure but still confirm low parent levels and rapid metabolism.	[48]

## Data Availability

All data is available in the manuscript.
